# Bioactive Compounds and Signaling Pathways of *Wolfiporia extensa* in Suppressing Inflammatory Response by Network Pharmacology

**DOI:** 10.3390/life13040893

**Published:** 2023-03-27

**Authors:** Juri Jin, Md. Helal Uddin Chowdhury, Md. Hafizur Rahman, Ki-Young Choi, Md. Adnan

**Affiliations:** 1 Division of Future Agriculture Convergence, College of Agriculture and Life Science, Kangwon National University, Chuncheon 24341, Republic of Korea; juri.jin@kangwon.ac.kr; 2 Ethnobotany and Pharmacognosy Lab, Department of Botany, University of Chittagong, Chattogram 4331, Bangladesh; helaluddinchowdhurycu@gmail.com; 3 Department of Bio-Health Convergence, College of Biomedical Science, Kangwon National University, Chuncheon 24341, Republic of Korea; hafizknu94@gmail.com

**Keywords:** *Wolfiporia extensa*, inflammation, HIF-1 signaling pathway, N-(3 chlorophenyl) naphthylcarboxamide, network pharmacology

## Abstract

*Wolfiporia extensa* (WE) is a medicinal mushroom and an excellent source of naturally occurring anti-inflammatory substances. However, the particular bioactive compound(s) and mechanism(s) of action against inflammation have yet to be determined. Here, we studied anti-inflammatory bioactive compounds and their molecular mechanisms through network pharmacology. Methanol (ME) extract of WE (MEWE) was used for GC-MS analysis to identify the bioactives, which were screened by following Lipinski’s rules. Public databases were used to extract selected bioactives and inflammation-related targets, and Venn diagrams exposed the common targets. Then, STRING and Cytoscape tools were used to construct protein-protein (PPI) network and mushroom-bioactives-target (M-C-T) networks. Gene Ontology and KEGG pathway analysis were performed by accessing the DAVID database and molecular docking was conducted to validate the findings. The chemical reactivity of key compounds and standard drugs was explored by the computational quantum mechanical modelling method (DFT study). Results from GC-MS revealed 27 bioactives, and all obeyed Lipinski’s rules. The public databases uncovered 284 compound-related targets and 7283 inflammation targets. A Venn diagram pointed to 42 common targets which were manifested in the PPI and M-C-T networks. KEGG analysis pointed to the HIF-1 signaling pathway and, hence, the suggested strategy for preventing the onset of inflammatory response was inhibition of downstream NFKB, MAPK, mTOR, and PI3K-Akt signaling cascades. Molecular docking revealed the strongest binding affinity for “N-(3-chlorophenyl) naphthyl carboxamide” on five target proteins associated with the HIF-1 signaling pathway. Compared to the standard drug utilized in the DFT (Density Functional Theory) analysis, the proposed bioactive showed a good electron donor component and a reduced chemical hardness energy. Our research pinpoints the therapeutic efficiency of MEWE and this work suggests a key bioactive compound and its action mechanism against inflammation.

## 1. Introduction

Inflammation is a crucial component of innate immunity’s protection against infections or tissue damage brought on by pathogen invasion, non-microbial stimuli, chemical stimulants, and toxicants, as well as by improper autoimmune reactions [1,2,3]. It may be distinguished by the presence of fluid accumulation and active cells, which are often symptoms of tissue degeneration [4]. Thus, the primary functions of inflammation are the elimination of pathogens and cell repair, but effective inflammation resolution and the restoration of homeostasis are essential for preventing inflammatory disorders [5,6]. Numerous variables, such as a shortage of antioxidants, vitamins, and anti-inflammatory substances such as zinc and selenium, might contribute to an insufficient inflammatory resolution. Reactive oxygen species (ROS) are formed during the inflammatory process as a consequence of the synthesis of cytokines and other pro-inflammatory mediators [7,8,9,10]. Hence, nonsteroidal anti-inflammatory drugs (NSAIDs) have been utilized as successful treatments for inflammatory disorders. However, these medications might cause serious gastrointestinal toxicity that is linked to thrombus formation, elevated blood pressure, and congestive heart failure [11,12,13]. Because of these drawbacks, there is a pressing need for innovative anti-inflammatory medicines originating from natural sources, notably plants and mushrooms, that have the ability to decrease the release of inflammatory mediators while also minimizing adverse side effects.

Mushrooms are valued functional foods used for their culinary values (taste and flavor) and medicinal (disease prevention) properties [14,15,16,17], notably hypocholesterolemic [18] and antiproliferative [19] effects. *Wolfiporia extensa* (Peck) Ginns (syn. *Poria cocos* F.A.Wolf) is a nutritious pharmaceutical mushroom, traditionally used to treat various ailments such as inflammation, acute gastroenteric catarrh, chronic gastritis, dizziness, edema, nausea, nephrosis, and gastric atony [20]. The primary metabolite from *W. extensa*, “Polysaccharide-II or 1,6-branched 1,3-alpha-D-galactan”, successfully delayed the production of the IFNγ activated IP-10 inflammation marker through regulating the expression of the IP-10 gene at the translational level while assuring human vascular endothelial cell (EC) toxicological safety, and also showed its efficiency as a new anti-inflammatory agent [21]. In mice, the colitis induced by TNBS was successfully alleviated by mitigating pro-inflammatory cytokines and boosting anti-inflammatory mediators in blood and colon-tissue by employing CMP33 from *W. extensa* [22]. Exploratory research concerning secondary metabolites revealed an anti-inflammatory action shown by ethanolic extract of *W. extensa* sclerotium on paw edema in acute and chronic stages [23]. Lee et al. deduced that six triterpenoids extracted from *W. extensa* sclerotia regulated NO and PGE2 levels in LPS-induced Raw 264.7 cells, downregulating iNOS and COX-2 expression [24,25]. Despite such evidence of W. extensa’s anti-inflammatory potential, there is no information on the cellular signaling mechanism of its therapeutic action or how it might exert anti-inflammatory effects to restore tissue damage or injury in individuals.

Hopkins first introduced Network Pharmacology in 2007 [26], an integrated drug discovery endeavor that includes system biology, bioinformatics and multidirectional pharmacology of the system [27]. This newly emerged process can pinpoint the molecular system of disease and the pathways of the biochemical framework of drugs at the molecular level [28,29]. It has changed the research paradigm from the “one target, one medication” model, to the newly postulated “multiple targets, multi-components” approach, by offering comprehensive composite target-compound and target-pathway networks to facilitate rationality and compatibility of medicines [30].

**Scheme 1:**
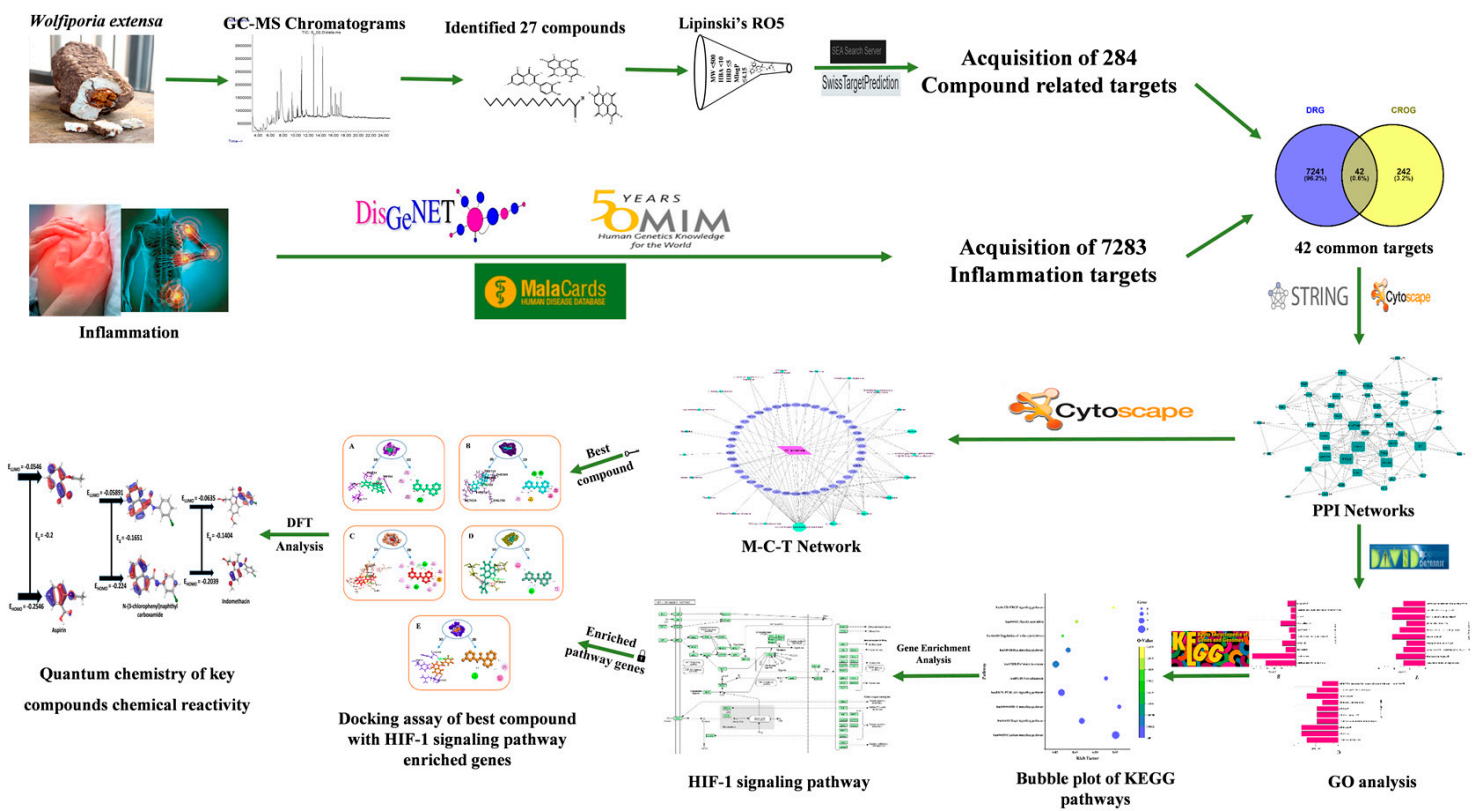
Schematic representation of network pharmacology approach for *W. extensa*.

In this study, a comprehensive network pharmacology approach was employed to explore the potential molecular mechanisms of the bioactive compounds of WE supporting its anti-inflammatory action. To identify bioactives from WE, gas chromatography and mass spectroscopy (GC-MS) analyses were conducted. Drug-likeness parameters were additionally filtered; the targets related to filtered bioactives and inflammation were acquired by accessing public databases. Common targets among them were culled, generating a substantial network to find core targets and bioactives. Gene set enrichment analysis was employed to unearth the targets linked to the hub signaling pathway, biological process, cellular component, and molecular function prediction. Finally, the selected targets were subjected to molecular docking simulation and quantum parameter analysis (DFT—Density Functional Theory) for extensive affirmation of WE’s chemical reactivity to determine the most effective anti-inflammation candidate (Scheme 1).

## 2. Materials and Methods

### 2.1. Mushroom Collection, Identification, and Extraction

The mushrooms (*Wolfiporia extensa*) were collected from (Latitude: 36. 666700, Longitude: 128. 510729, Gyeongsangbuk-do, Republic of Korea) in December 2020. The collected mushrooms (200 g) were dried at ambient temperature (20–22 °C; for 7 days) and ground into a coarse powder using an automated grinder. The refined powder (30 g) was soaked in 300 mL of methanol (Daejung, Siheung, Republic of Korea). The extraction (repeated 3 times at room temperature) was carried out in a sealed bottle, with continuous shaking and stirring (for 5 days) using an electric shaker machine in order to increase the yield rate. The mixture was filtered (Whatman qualitative filter paper Grade 1) and evaporated using a vacuum evaporator (IKA, Staufen, Germany). The evaporated sample (MEWE) was dried using a hot water bath (IKA, Staufen, Germany) at 40 °C and preserved in a refrigerator (−4 °C) for GC-MS analysis.

### 2.2. GC-MS Analysis

MEWE was analyzed by GC-MS technique using the GC-MS system (Agilent 7890A, 5975C Agilent Technologies Inc., Santa Rosa, CA, USA) and a DB-5MS capillary column (30 m × 0.25 µm × 0.25 mm). The detailed protocol was described in our previous study [31].

### 2.3. Bioactive Compounds Filtration

The bioactive compounds (by GC-MS) were detected by the drug-likeness protocol “Lipinski’s rule of five” to obtain potentially active compounds with a drug-like character. Each compound was considered in terms of the absorption, distribution, metabolism and excretion (ADME) framework and the requirement for an oral bioavailability score > 0.50. Here, the essential pharmacokinetic factor is the oral bioavailability (OB) aspect of the ADME processes [32]. This analysis was performed through accessing an online tool, Swiss ADME [33]. SMILES notations for the compounds were obtained from PubChem (https://pubchem.ncbi.nlm.nih.gov/, accessed on 23 December 2021) database.

### 2.4. Extraction of Compound Associated Targets and Inflammatory Targets

Using ‘Homo sapiens’ mode, we compiled the target genes associated with filtered bioactives by entering their SMILES into the SEA (Similarity Ensemble Approach) (http://sea.bkslab.org/, accessed on 7 January 2022) and STP (Swiss Target Prediction) (http://www.swisstargetprediction.ch/, accessed on 9 January 2022) databases. In contrast, inflammation-related genes were gathered accessing DisGeNeT (https://www.disgenet.org/search, accessed on 18 January 2022) [34], Malacards (https://www.malacards.org/, accessed on 24 January 2022) [35] and OMIM (https://www.ncbi.nlm.nih.gov/omim, accessed on 5 February 2022) [36] databases. VENNY 2.1 (https://bioinfogp.cnb.csic.es/tools/venny/, accessed on 13 February 2022) displayed the common targets among MEWE bioactives and inflammatory target genes.

### 2.5. Common Targets Network Construction

To assess possible protein interactions, the common targets were entered into the String Database (https://string-db.org/, accessed on 25 February 2022) in ‘Homo sapiens’ mode, with a medium confidence level of 0.400. For better visualization, we imported the network into Cytoscape 3.8.2 software [37], and the whole network was analyzed in Cytoscape using the CytoHubba plugin and the following three algorithms: Maximal Clique Centrality (MCC) [38], Maximum Neighborhood Component (MNC), and Degree value [39]. Formulas are given below:

MCC(v) = ∑C∈S(v) (|C| − 1)! (1)

where S(v) is the set of maximum cliques containing v and (|C| − 1)! is the sum of all positive integers that are smaller than |C|.

MNC(v) = |V(MC(v))|(2)

where MC(v) is the G[N(v)]’s mostly linked component and G[N(v)] is the G’s induced subgraph by N(v).

Deg(v) = |N(v)|(3)

where N(v) represents the connections of v’s neighbors, and v is their respective node.

### 2.6. Mushroom-Bioactives-Targets Network Construction

The bioactive compounds of MEWE, common inflammatory targets, and mushroom were loaded into Cytoscape 3.8.2 software to generate a graphical representation of the Mushroom-Compound-Target network. The merging function plugin Cytoscape was used to create this network. The Network Analyzer was used to evaluate network topology parameters. Nodes represent bioactives, targets, and mushrooms, and edges indicate their interaction. The frequency of connective neighbors of a node is referred to as its degree. The greater the number of linked edges in a node, the greater the impact [40].

### 2.7. Analysis of GO and KEGG Pathway Involvement within Common Targets

All common targets were submitted to the Annotation, Visualization, and Integrated Discovery (DAVID, https://david.ncifcrf.gov/tools.jsp, accessed on 10 March 2022) database for molecular functional annotation and KEGG pathway analysis to uncover their involvement in signal transduction. We chose OFFICIAL GENE SYMBOL as the identifier and Homo sapiens as the species for this enrichment analysis. In network pharmacology, the KEGG database has immense significance in illustrating targets involved in a disease’s molecular mechanism. The GO database depicts the biological descriptors of those targets, notably, Biological Process (BP), Cellular Component (CC) and Molecular Function (MF) [41]. A threshold value for GO terms and pathways enrichment was selected as *p*-value < 0.05. The FDR error control approach was used to correct the *p*-value, and the outcome was referred to as the Q value. The bubble plot map of KEGG pathways was graphically displayed utilizing Origin Pro 2021 to analyze the pathways. 

### 2.8. Preparation of Ligand and Receptor Protein

Preferential ligands revealed from compound-target network research included conventional drugs such as Aspirin and Indomethacin; co-crystallized protein ligands were acquired in .sdf format using the PubChem chemical library. Furthermore, the metabolites were processed for molecular docking assays using the LigPrep program included in the Schrödinger suite-Maestro v12.5, adopting the previously reported techniques [42]. In addition, 5 receptor proteins of the hub signaling pathways targets with crystal structures had been selected for accessing in the RCSB Protein Data Bank (https://www.rcsb.org/, accessed on 19 March 2022) and the UniProt database (https://www.uniprot.org/, accessed on 19 March 2022), within which each of them have been accessible, i.e., TLR4 (PDB ID: 3UL7), EGFR (PDB: 5WB7), FLT1 (PDB: 3HNG), NOS3 (PDB: 1M9J) and NOS2 (PDB: 1NSI). The Schrödinger Suite-Maestro v12,5 embedded Protein Preparation Wizard tools have been configured once the 3D crystal structure have been found from the RCSB database following our previously described protocols [43,44].

### 2.9. Molecular Docking Simulation Using Glide

We utilized Glide tools plugged-in Schrödinger Suite-Maestro version 12.5 software for molecular docking investigations to identify receptor grids’ active site molecules (co-crystallized ligand site) [42]. During grid preparation, we employed default topological parameters such as the vdw (van der Waals) scaling factor 1.00, the OPLS3 force field and charge cut-off value 0.25 for individual 3D protein structures. At the plausible docking site, a cubic box of specific dimensions was set on kernel active site residues of macromolecules and the box size was given 14 Å × 14 Å × 14 Å co-ordinates. The docking experiments were subsequently implemented deploying the standard precision (SP) scoring algorithm of Glide, with each ligand noted individually in terms of the highest rating posture and docking score.

### 2.10. Quantum Chemistry of Frontier Molecular Orbitals

By following the Lee Yang Parr (B3LYP-D3) correlation functional approach at the 6–31G++ (d,p) level basis set, all compounds’ (hub and standard drugs) structural coordinates were thoroughly optimized using Jaguar panel of Maestro 12.5 software [45]. This optimized geometry also yielded frontier molecular orbital energies of HOMO (highest occupied molecular orbital) and LUMO (lowest occupied molecular orbital). In order to compute the HOMO-LUMO gaps of each chemical, LUMO energy was subtracted from the appropriate HOMO energy value. Depending on the energies of frontier HOMO and LUMO, the following formulae computed each compound’s hardness (ɳ) and softness (S) to hypothesize their chemical reactivity.

ɳ = (HOMO − LUMO)/2

S = 1/ɳ

## 3. Results

### 3.1. Chemical Composition of MEWE

**Figure 1:**
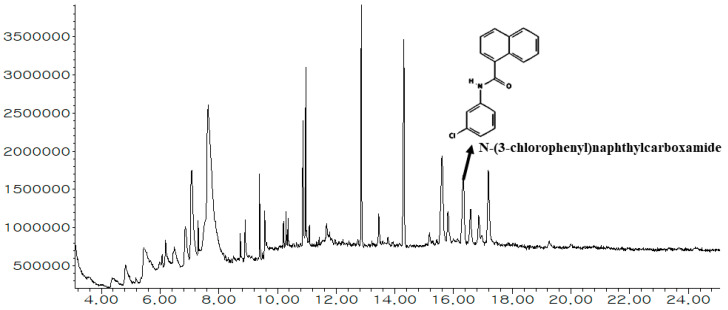
GC-MS peak of the methanolic extract of *W. extensa* and an indication of the key bioactive.

Gas Chromatography-Mass Spectrometry (GC-MS) analysis revealed the presence of 27 bioactives in MEWE (Figure 1). The spectrometric data of identified bioactives, including RT (Retention Time), area (%), chemical formula and the names of bioactives, are displayed in Table 1. Several diversified chemical classes including Organooxygen compounds, Pyrans, Acyl halides, Isothiocyanates, Azolines, Boronic acid derivatives, Carboxylic acids and derivatives, Fatty acyls, Naphthalenes, Benzene and substituted derivatives, Pyrimidine nucleosides, Glycerolipids, Organonitrogen compounds, and Steroids and steroid derivatives were found in MEWE.

### 3.2. Bioactive Compounds Filtration

Following Lipinski’s rule, the bioactives which have a molecular weight not more than 500, hydrogen bond acceptor (HBA) not more than 10, hydrogen bond donor (HBD) not exceeding 5, Moriguchi octanol-water partition coefficient score not exceeding 4.15 and maintaining a standard ‘Abott Bioavailability Score’ of not more than 0.1 were considered as biologically active constituents. Following these filtration criteria, all bioactives (27) showed drug likeliness while not violating more than one of Lipinski’s assertions (Supplementary File S1: Table S1). 

### 3.3. Common Targets of Bioactives Intersected from SEA and STP Databases

**Figure 2:**
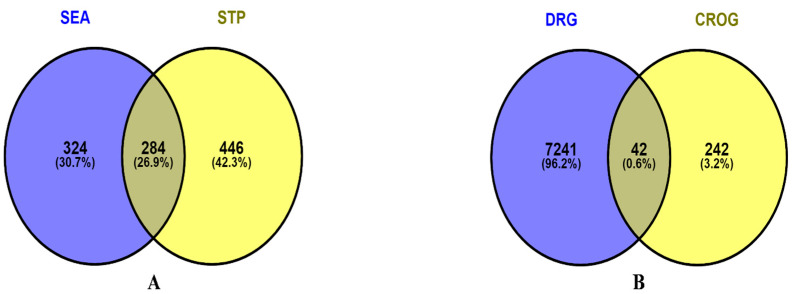
Intersecting targets. (**A**) Bioactives related targets from SEA and STP; (**B**) Inflammation targets from DisGeNet, Malacards and OMIM, and common bioactives-related targets.

After filtration, bioactives-associated targets were extracted from the SEA and STP databases. Inputting bioactives-specific SMILES in the respective databases, a total of 608 targets from SEA and 730 targets from STP were gathered after removing duplication (File S2). Finally, Venn analysis revealed 284 common targets (File S3) between two public databases (Figure 2A). 

### 3.4. Identification of Inflammation Targets within Disease Targets and 284 Common Targets

Three public databases, namely, DisGeNET, OMIM, and Malacards, yielded a total of 7283 targets associated with inflammation (File S4). The Venn diagram showed 42 common targets (File S5) when comparing the inflammatory targets (7283) and 284 bioactives-related targets (Figure 2B). However, the identified 42 common targets were connected to 21 out of the 27 bioactives. No targets were found in either database that connected with the remaining 6 bioactives, namely, 2,4-Dimethyl-1,3-cyclopentanedione; 3-Hydroxy-2,3-dihydromaltol; 2-Butynol; 2-Butenoyl chloride; alpha-Hydroxyisobutyric acid cyclic butaneboronate and N4 hydroxycytosine.

### 3.5. Network Construction of 42 Common Targets

The extracted 42 common targets were added to the STRING database to generate a PPI network to uncover possible mechanisms of MEWE anti-inflammatory action. The STRING network analysis indicated that each target in the network was related to the others via 42 nodes, 177 edges, and an average number of neighbors of 8.429, where network diameter and radius were 4 and 2, respectively. The Cytoscape network was used to explore key targets in the network, by means of three algorithms (MCC, MNC and Degree value) integrated with cytoHUBBA to increase network node precision and accuracy. Interestingly, TLR4, EGFR, and NOS3 were the top three essential targets in all algorithm-based networks studied (File S1: Figure S1). Table 2 shows the ranking of each gene present in the network according to the three algorithms.

### 3.6. Analysis of Mushroom-Compound-Targets Network

**Figure 3:**
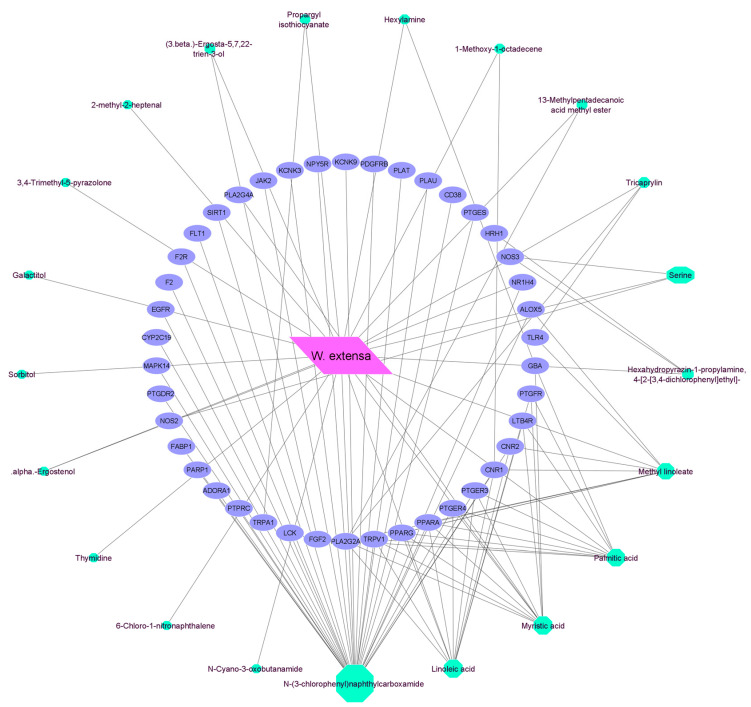
Bioactives—Target network interaction between WE bioactives and common inflammation targets.

The mushroom-compound-targets network was constructed to evaluate the interconnection between mushrooms components and common inflammatory targets. The Network Analyzer applications in Cytoscape ascertained that 100 edges were bound to 64 nodes in the network; 42 nodes were common disease targets, 21 nodes referred to compounds and one node referred to the mushroom, viz. W.extensa (Figure 3). To detect key chemicals within the network, compounds that interacted with targets and mushrooms were assessed based on their relationship with adjacent targets, referred to as the ‘Degree Value’. Finally, N-(3-chlorophenyl)naphthylcarboxamide was exposed as the most active and potent metabolite in the network, which might protect against inflammation [File S1: Table S2].

### 3.7. Gene Ontology (GO) Analysis of 42 Common Targets

Gene ontology (GO) enrichment assessment of potential targets was performed to elucidate the crucial pharmacological processes as well as to corroborate the biological features (biological processes, chemical contents and molecular function) of the 42 inflammation targets. Here, the first ten functional keywords were picked based on gene percentages. File S1: Figure S2A demonstrates that the top 10 biological processes (BP) were the release of sequestered calcium ions into the cytosol, response to lipopolysaccharide, positive regulation of phosphatidylinositol 3-kinase signaling, negative regulation of blood pressure, inflammatory response, positive regulation of ERK1 and ERK2 cascade, activation of MAPKK activity, negative regulation of the apoptotic process, positive regulation of cell proliferation and positive regulation of cytosolic calcium ion concentration. The top 10 chemical contents (CC) were the integral component of the plasma membrane, plasma membrane, membrane raft, cell surface, an intrinsic component of the plasma membrane, nuclear envelope, extracellular space, caveola, and the extrinsic component of the cytoplasmic side of plasma membrane focal adhesion (File S1: Figure S2B). The molecular function (MF) output also indicated the presence of protein tyrosine kinase activity, enzyme binding, receptor binding, phosphatidylinositol-4,5-bisphosphate 3-kinase activity, protein phosphatase binding, drug binding, phosphatidylinositol 3-kinase binding, protein kinase binding, Ras guanyl-nucleotide exchange factor activity and RNA polymerase II transcription factor activity and ligand-activated sequence-specific DNA binding characteristics, which may contribute to the anti-inflammatory activity of MEWE (File S1: Figure S2C).

### 3.8. KEGG Pathway Enrichment Analysis of Identified 42 Common Targets

**Figure 4:**
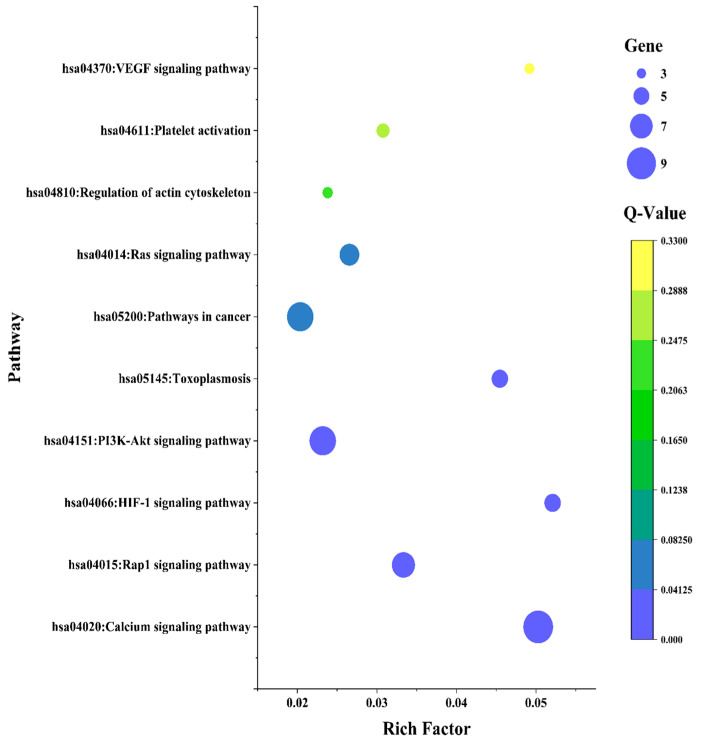
Bubble plot of significantly enriched pathways of common targets in KEGG evaluation.

**Figure 5:**
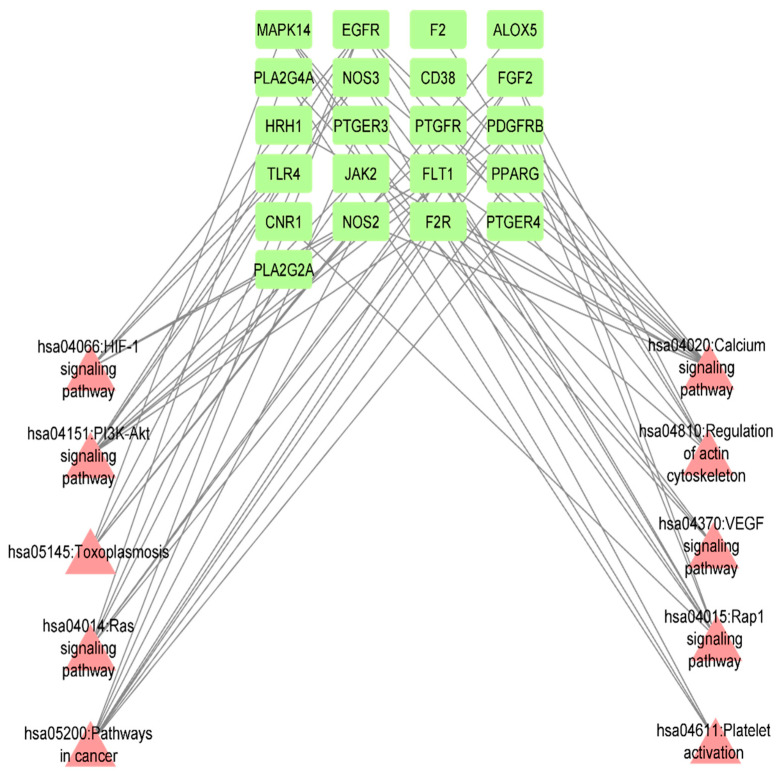
Pathway-Gene network of common targets.

**Figure 6:**
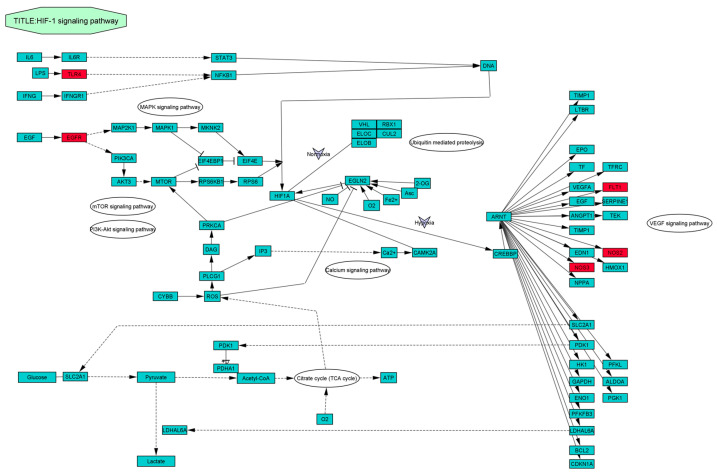
Illustration of HIF-1 signaling pathway. The red node represents the enriched targets in the present study.

Data in File S1: Table S3 show that 21 of the 42 common targets were actively engaged in inflammation progression connected to 10 KEGG (Kyoto Encyclopedia of Genes and Genomes) annotation pathways (filtered at less than 0.05 *p*-value). In order to undertake the pathway enrichment analysis, the rich factor and corrected False Discovery Rate (FDR) value (Q-value) were considered. The rich factor shows the extent of the pathway enrichment with a significantly lower Q value. Figure 4 elucidates that the HIF-1 signaling pathway was the most enriched within the selected targets in conformity with prior filtration. Importantly, the Target-Pathway Network in Figure 5 provides intrinsic insight into targets and the pathway relationships. Details of the core pathway, namely the HIF-1 signaling pathway, are shown in Figure 6.

### 3.9. Docking Score Assessment of Key Bioactives and 5 HIF-1 Signaling Pathway Targets

The compound-target network analysis identified “N-(3-chlorophenyl)naphthylcarboxamide” as the best bioactive component. It was therefore investigated to evaluate its binding affinity with five targets of the HIF-1 signaling pathway linked to inflammatory function (Table 3). The co-crystallized ligands of macromolecules were re-docked to assess complex binding stability energy and compare disease reaction to a typical medication (Aspirin, Indomethacin) for further validation. 

**Figure 7:**
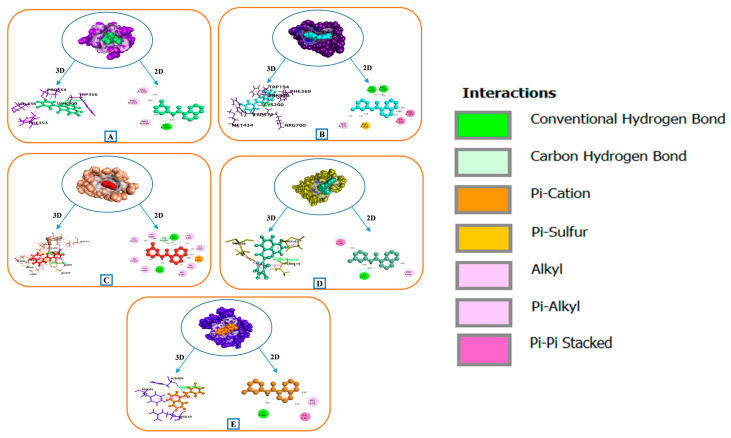
Binding relationship between targets enriched in the HIF-1 signaling pathway and the key bioactive “N-(3-chlorophenyl)naphthylcarboxamide” from MEWE. (**A**) 1M9J (**B**) 1NSI (**C**) 3HNG (**D**) 3UL7 and (**E**) 5WB7.

In terms of docking affinity, N-(3-chlorophenyl)naphthylcarboxamide connecting to TRP-356, VAL-336, PHE-353, and PRO-334 residues (forming one hydrogen and three hydrophobic bonds) of 1M9J exposed a substantial impact on the linkage with NOS3 (PDB ID: 1M9J) (Figure 7A). Compared to conventional medicines such as Aspirin (−5.262 Kcal/mol) and Indomethacin (−7.451 kcal/mol), the affinity of the interaction complex (bioactive-NOS3) was −7.236 kcal/mol. 

An interface complex of N-(3-chlorophenyl)naphthylcarboxamide with NOS2 (PDB ID: 1NSI) was stabilized by the 2-H bond (CYS-200 and ARG-700) and 10 hydrophobic (TRP-194, PHE-369, MET-434, TRP-372, and CYS-200) and one pi-sulfur (MET-434) interactions. This interaction revealed an optimal docking energy of −8.85 kcal/mol as compared to the reference medicine Aspirin (−6.467 kcal/mol) and Indomethacin (−7.491 kcal/mol) (Figure 7B). 

The binding between the bioactive and the active site of FLT1 (PDB ID: 3HNG) target demonstrated −8.88 kcal/mol energy by three H-bonds (ASP-1040, GLU-878, and CYS-1039), ten hydrophobic bonds (VAL-891, LEU-1013, ILE-1038, VAL-841, ALA-859, LYS-861, VAL-892, VAL-909, and LEU-882), two electrostatic bonds (LYS-861) and one pi-sulfur bond (LYS-1039). This energy was substantially lower than for the conventional medicine Aspirin and Indomethacin, with energy values of −6.794 kcal/mol and −5.538 kcal/mol, respectively (Figure 7C). 

The TLR4 gene (PDB ID: 3UL7) exhibited the comparable binding affinity with the bioactive (−4.027 kcal/mol), through one hydrogen bond of LEU-119 residues, three hydrophobic bonds of HIS-148, PRO-145, and LEU-119 residues (Figure 7D), which was a contrast to standard medications Aspirin (−4.178 kcal/mol) and Indomethacin (−4.168 kcal/mol). 

In the 5WB7 (EGFR target) scenario, interaction with the bioactive revealed that the active pocket included one H-bonding with HIS-409 and two H-phobic bondings with PHE-45 and ARG-29 residues (Figure 7E). The resulted docking score (−4.123 kcal/mol) was almost parallel to the competitive standards Aspirin (−4.182 kcal/mol) and Indomethacin (−4.707 kcal/mol). Details of binding information are given in File S1: Table S4.

However, this best bioactive “N-(3-chlorophenyl)naphthylcarboxamide” interaction with TLR4 and EGFR targets displayed almost equivalent binding affinity relative to its co-crystallized complex energy. Aside from that, the bioactive complex with FLT1 and NOS2 showed relatively low docking energy, unlike the respective co-crystallized ligands; values <−8 kcal/mol indicate significantly identical stability energy for any complex. However, the complex with the NOS3 target had greater affinity than the co-crystallized ligands. 

### 3.10. DFT Evaluation of Key Compound and Standard Drugs

**Figure 8:**
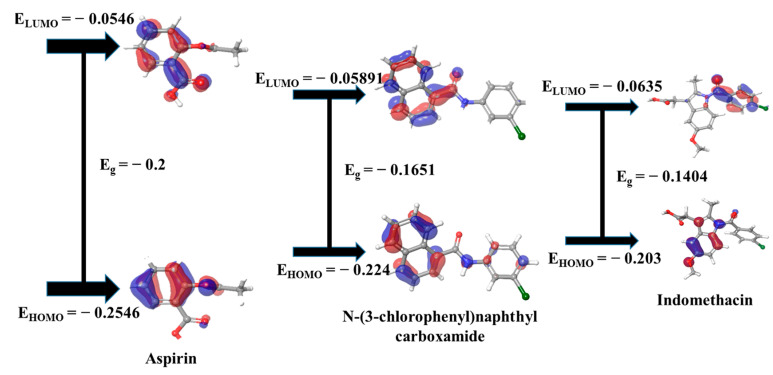
Frontier Molecular Orbital plots and their energy for key compounds from MEWE and standard medicines.

The DFT (Density Functional Theory) of key compounds and standard drugs was checked to explore their chemical reactivity with other species or targets. Generally, how a chemical molecule donates or accept its valence electrons to the ligand can be confirmed by the HOMO and LUMO levels. Here, the key bioactive “N-(3-chlorophenyl)naphthyl carboxamide” showed a considerable HOMO energy (−0.224 Kcal/mol) and appears to be a good electron donor constituent compared to the standard drugs, Indomethacin and Aspirin. The stability of a compound depends on the HOMO-LUMO energy gap, and lower energy is linked to a soft molecule. The key bioactive manifested a lower chemical hardness energy (0.08255 Kcal/mol) than standard drug Aspirin (File S1: Table S5). Figure 8 displays the frontier molecular orbitals localization pattern of the ground state (HOMO) and the first excited state (LUMO) of corresponding compounds.

## 4. Discussion

From the pathophysiological point of view, inflammatory disorders are complex, including a cascade of events that may result in severe sickness and include several proteins and pathways. Herbal treatments have long been used as an integral part of standard medicine practice due to their rich chemical components [46]. However, in most situations, the pharmacological mechanism of action of traditional medicines is still unknown. Network pharmacology, in this regard, provides a fresh perspective on the search for effective herbal substances against various diseases [47].

Through utilizing the GC-MS technique, a total of 27 bioactives from MEWE were screened. Of them, 21 out of 27 bioactives were potentially directly involved in the therapeutic efficiency of MEWE against inflammation. In addition, the compound-target network exposed 42 inflammatory targets intimately associated with 21 bioactives in the mechanism of inflammation. Among them, N-(3-chlorophenyl)naphthylcarboxamide was classified as a core essential bioactive in the network. In addition, the KEGG pathway enrichment analysis of 42 common targets disclosed that the BP, CC and MF activities interacted with 10 signaling pathways, among which 8 signaling pathways were closely related to the inflammation process and development. The causality of the 10 signaling pathways in inflammation are outlined here.

VEGF signaling pathway: VEGF activation induces angiogenesis in rheumatoid arthritis, which increases synovium nutrient flow, leukocyte motility, and cytokine release. Angiogenic factors make tumors more vascular, leading to a faster spread [48,49]. Platelet activation pathway: activated platelets produce IL-6, IL-8, IL-1, and TNF- α (insoluble versions) and regulate pro-inflammatory actions such as phagocytosis, leukocyte migration, and ROS generation. These mediators affect vascular inflammation, asthma, atherosclerosis, and rheumatoid arthritis [50]. Regulation of actin cytoskeleton: immunodeficiency or autoinflammatory diseases are linked to protein scrappiness (actin severing proteins, nucleation promoting factors, stabilizing protein of actin, de-polymerizing protein of actin and actin nucleators). These proteins subsequently intertwine with the actin cytoskeleton [51]. Ras signaling pathway: Ras activation produces pro-inflammatory cytokines, contributing to rheumatoid arthritis and vascular inflammation [52]. Pathways in cancer: intrinsic and extrinsic processes connect cancer with inflammation, activating transcription factors including NFKB, STAT-3, and HIF-1. These variables cause tumor cell growth. Thus, tumor-associated inflammation rises, halting immunological defenses [53]. Toxoplasmosis: parasite effectors may slow IFN-triggered toxoplasmacidal processes, causing toxoplasma-induced inflammation. Such effectors affect STAT3/6 (upregulated by ROP16), NFKB (upregulated by GRA15), and MAPK (induction by ROP38) signaling pathways that impact cytokine production [54]. PI3K-Akt signaling pathway: cytokine TNF-α induced phosphatidylinositol-3-kinase and its downstream target Akt stimulation lead to the phosphorylation of IKK, which activates NFKB, and subsequently induces vascular diseases [55]. HIF-1 signaling pathway: hypoxia-activated NFkB stimulates the synthesis of pro-inflammatory cytokines and growth factors via HIF signaling pathways, causing hyperglycemia, cancer, atherosclerosis, and rheumatoid arthritis [56]. Rap1 signaling pathway: macrophage Rap1 enhanced IKB and p65 phosphorylation, allowing NFkB binding to DNA kB sites and influencing pro-inflammatory gene transcription. Rap1 in inflammatory macrophages may promote atherosclerosis [57]. Calcium signaling pathway: channels in endothelial cells are opened when inflammatory mediators such as vasoactive amines, peptides, protease thrombin, and eicosanoids interact with receptors on these cells [58]. 

**Figure 9:**
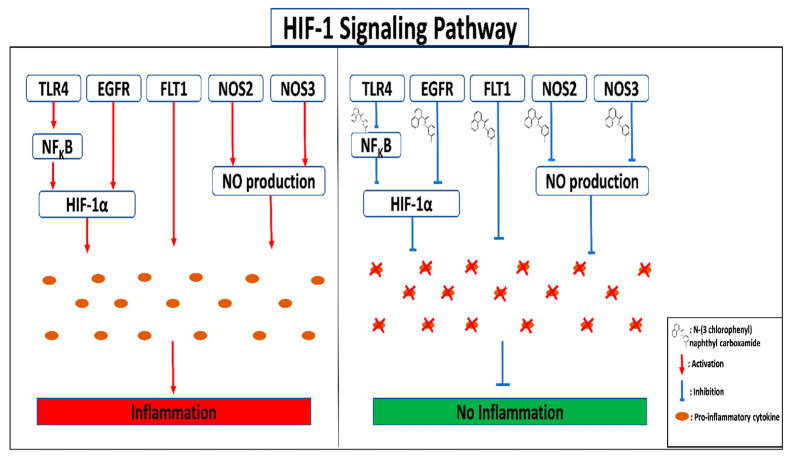
Mechanistic mode of action of *W. extensa* against inflammation.

These 10 signaling pathways were shown to have direct involvement in the initiation of inflammation. However, there was a much higher enrichment level for the HIF-1 signaling pathway than for other similar rich factors. Significantly, the largest enrichment occurs at the highest rich factor [59]. In a mechanistic sense, the hypoxia caused a significant reduction in the activity of the HIF hydroxylase enzyme. In addition, HIF activation by TLRs and the EGFR downstream channel stabilized HIF, allowing dimerization with HIF-1 and attachment to p300 co-activators following nucleus accumulation. In addition, hypoxia-induced downregulation of IKK2 results in phosphorylation and degradation of IkB and activation of NFkB. The sensitivity of several variables involved in angiogenesis, nitric oxide synthase, and inflammation is influenced by HIF signaling because of this enriched and downstream route [60]. Ultimately, “TLR4” controls the synthesis of inflammatory cytokines via regulating the activation of the NFkB pathway’s RelA/p50 transcription factor complexes [6]. Consistently activated NFkB perpetuates an invasive phenotype by upregulating cell cycle regulators, anti-apoptotic, proteolytic factors, and pro-inflammatory cytokines [53]. The EGFR gene also activates downstream pathways, including the MAPK and PI3K-Akt signaling pathways that regulate HIF-1 upon hypoxia. Nitric oxide (NO) production caused by the activation of nitric oxide synthases (NOS2) triggers several pre-cancerous and malignant lesions, such as Barrett’s mucosa [61]. In addition, the aberrant expression of the iNOS/eNOS enzymes can induce inflammation-related cardiomyocyte mortality and protein nitration disorder [62]. The elevated expression of FLT1 may also pertain to the development of rheumatoid arthritis inflammation [63]. These findings support the hypothesis that the HIF-1 signaling pathway is associated with inflammatory illnesses such as rheumatoid arthritis, inflammatory bowel disease, chronic renal disease, atherosclerosis, and asthma [64]. As a result, inhibiting NFKB activation and HIF-1’s downstream pathways may be a viable therapeutic strategy for treating inflammation (Figure 9). Five targets in the HIF-1 signaling pathway were rigorously docked with the essential molecule discussed here to ascertain the sensitivity of this pathway.

The docking assay concluded that FLT1, NOS3 and NOS2 demonstrated superior binding interaction to the key essential component N-(3-chlorophenyl)naphthyl carboxamide compared to either reference medicine (Aspirin, Indomethacin). TLR4 and EGFR transcribed protein’s binding energy and stability were almost identical to Aspirin, Indomethacin and co-crystallized ligands. Conversely, FLT1 and NOS2 had indistinguishable compound docking complex stability in response to corresponding co-crystallized docking affinity, but NOS3 had the best stability. Quantum chemical analysis at the DFT (Density Functional Theory) level has confirmed the compound’s chemical reactivity. The kinetic stability and chemical reactivity of a molecule are largely determined by its HOMO and LUMO energy gaps [65]. The high kinetic stability and low chemical reactivity may be attributed to a sizable HOMO-LUMO gap which helps to explain the chemical function descriptors like hardness and softness [66,67,68]. The softness of a chemical boosts the reactivity and our explored hub compound “N-(3-chlorophenyl)naphthyl carboxamide” demonstrated a greater degree of softness energy than Aspirin, suggesting that the compound had strong binding affinity to the targets. Overall, these findings imply that N-(3-chlorophenyl)naphthyl carboxamide could block key molecular targets that support inflammatory escalation.

## 5. Conclusions

In conclusion, we used a computer-assisted network pharmacology prediction to explore the molecular pathway process by which MEWE can act against inflammation, in order to learn more about how the HIF-1 signaling pathway and other important pathways influence inflammation. The docking simulation showed that “N-(3-chlorophenyl)naphthyl carboxamide” successfully inhibited hypoxia-induced HIF-1α activation through downregulation of downstream NFKB, MAPK, mTOR, and PI3K-Akt signaling channels related to inflammation in the HIF-1 signaling pathway. The low HOMO-LUMO energy gap of the compound confirmed its robust chemical reactivity behavior with potential targets. In spite of this, more pharmacodynamic and mechanistic research studies are required in order to obtain a complete understanding of the intricate synergistic activities that unite the pharmacological effectiveness of WE on inflammation, as described in this work.

## Figures and Tables

**Table 1:** A list of 27 GC-MS-detected chemical components in methanolic extract of *W. extensa*.

S.N.	R.T. (min)	Area (%)	PubChem CID	Chemical Formula	Bioactives Class	Bioactives Name
1	4.38	0.43	54544338	C_5_H_6_N_2_O_2_	Organooxygen compounds	N-Cyano-3-oxobutanamide
2	4.43	0.8	580975	C_7_H_10_O_2_	Organooxygen compounds	1,3-Cyclopentanedione, 2,4-dimethyl- or 2,4-Dimethyl-1,3-cyclopentanedione
3	4.83	1.76	119838	C_6_H_8_O_4_	Pyrans	2,3-Dihydro-3,5-dihydroxy-6-methyl-4h-pyran-4-one or 3-Hydroxy-2,3-dihydromaltol
4	5.45	4.58	12991	C_4_H_6_O	Organooxygen compounds	2-Butyn-1-ol or, 2-Butynol
5	5.68	0.74	643131	C_4_H_5_ClO	Acyl halides	(2E)-2-Butenoyl chloride or 2-Butenoyl chloride
6	5.75	0.82	123411	C_4_H_3_NS	Isothiocyanates	Thiocyanic acid, 2-propynyl ester or Propargyl isothiocyanate
7	5.99	0.99	76665	C_6_H_10_N_2_O	Azolines	3H-Pyrazol-3-one, 2,4-dihydro-4,4,5-trimethyl- or 3,4-Trimethyl-5-pyrazolone
8	6.08	0.68	5362763	C_8_H_14_O	Organooxygen compounds	2-Heptenal, 2-methyl- or 2-methyl-2-heptenal
9	6.2	1.96	8102	C_6_H_15_N	Organooxygen compounds	1-Hexanamine or Hexylamine
10	6.49	2.41	538272	C_8_H_15_BO_3_	Boronic acid derivatives	Lactic acid, 2-methyl-, monoanhydride with 1-butaneboronic acid, cyclic ester or alpha-Hydroxyisobutyric acid cyclic butaneboronate
11	6.87	2.88	11850	C_6_H_14_O_6_	Organooxygen compounds	Galactitol
12	7.08	7.14	5951	C_3_H_7_NO_3_	Carboxylic acids and derivatives	L-Serine or Serine
13	7.31	0.8	5366263	C_19_H_38_O	Organooxygen compounds	Ether, methyl 1-octadecenyl or 1-Methoxy-1-octadecene
14	7.35	0.41	11005	C_14_H_28_O_2_	Fatty acyls	Tetradecanoic acid or Myristic acid
15	7.64	24.3	5780	C_6_H_14_O_6_	Organooxygen compounds	Sorbitol
16	8.74	0.41	554151	C_17_H_34_O_2_	Fatty acyls	Pentadecanoic acid, 13-methyl-, methyl ester or 13-Methylpentadecanoic acid methyl ester
17	8.9	1.21	985	C_16_H_32_O_2_	Fatty acyls	Hexadecanoic acid or Palmitic acid
18	9.39	1.27	5284421	C_19_H_34_O_2_	Fatty acyls	9,12-Octadecadienoic acid (Z,Z)-, methyl ester or Methyl linoleate
19	9.57	1.33	5280450	C_18_H_32_O_2_	Fatty acyls	9,12-Octadecadienoic acid (Z,Z)- or, Linoleic acid
20	10.2	1.12	610075	C_10_H_6_ClNO_2_	Naphthalenes	Naphthalene, 6-chloro-1-nitro- or 6-Chloro-1-nitronaphthalene
21	10.3	1.07	581332	C_15_H_23_Cl_2_N_3_	Benzene and substituted derivatives	Hexahydropyrazin-1-propylamine, 4-[2-[3,4-dichlorophenyl]ethyl]-
22	11	3.29	5789	C_10_H_14_N_2_O_5_	Pyrimidine nucleosides	Thymidine
23	12.9	4.96	10850	C2_7_H_50_O_6_	Glycerolipids	Glycerol tricaprylate or Tricaprylin
24	14.3	6.43	159892	C_4_H_5_N_3_O_2_	Organonitrogen compounds	2-Hydroxy-4-hydroxylaminopyrimidine or, N4-hydroxycytosine
25	15.6	5.75	6432563	C_28_H_44_O	Steroids and steroid derivatives	Ergosta-5,7,22-trien-3-ol, (3.beta.,22E)- or (3.beta.)-Ergosta-5,7,22- trien-3-ol
26	16.3	4.24	532200	C_17_H_12_ClNO	Naphthalenes	1-Naphthalenecarboxamide, N-(3-chlorophenyl)- or N-(3-chlorophenyl)naphthylcarboxamide
27	16.6	1.8	312796	C_28_H_48_O	Steroids and steroid derivatives	.alpha.-Ergostenol

Notes: S.N. = Serial number; R.T. = Retention time.

**Table 2:** Classified orders of PPI network of 42 common targets based on different algorithms.

MCC			MNC			Degree		
Rank	Genes	Score	Rank	Genes	Score	Rank	Genes	Degree
1	TLR4	1298	1	TLR4	18	1	TLR4	18
2	EGFR	1232	1	EGFR	18	2	EGFR	18
3	NOS3	1180	3	NOS3	16	3	NOS3	16
4	PTPRC	1158	3	PTPRC	16	4	PTPRC	16
5	MAPK14	1085	5	F2	15	5	MAPK14	15
6	PPARG	969	6	MAPK14	14	6	F2	15
7	FGF2	920	7	TRPV1	13	7	TRPV1	13
8	JAK2	217	8	FGF2	12	8	FGF2	12
9	NOS2	148	9	ALOX5	11	9	ALOX5	11
10	CNR1	146	10	CNR1	10	10	PPARG	11
11	SIRT1	142	10	PPARG	10	11	CNR1	10
12	CNR2	134	10	PTGER4	10	12	PTGER4	10
13	ADORA1	132	10	SIRT1	10	13	SIRT1	10
14	PTGER3	130	14	PTGER3	9	14	PTGER3	9
15	PTGDR2	122	14	PTGES	9	15	PTGES	9
16	NPY5R	121	16	CNR2	8	16	JAK2	9
17	LCK	96	16	NOS2	8	17	CNR2	8
18	PTGER4	84	16	JAK2	8	18	PLA2G4A	8
19	TRPV1	82	16	PLA2G4A	8	19	LTB4R	8
20	F2	72	20	ADORA1	7	20	F2R	8
21	PDGFRB	54	20	HRH1	7	21	NOS2	8
21	ALOX5	54	20	LCK	7	22	ADORA1	7
23	PTGES	38	20	TRPA1	7	23	PTGDR2	7
24	FLT1	36	20	F2R	7	24	LCK	7
25	F2R	33	25	PDGFRB	6	25	TRPA1	7
26	PLA2G4A	32	25	FLT1	6	26	PPARA	7
26	HRH1	32	25	PLAU	6	27	HRH1	7
26	CD38	32	25	CD38	6	28	NPY5R	6
29	LTB4R	30	29	PTGDR2	5	29	CD38	6
30	PTGFR	26	29	NPY5R	5	30	PDGFRB	6
31	PLAU	24	29	PARP1	5	31	FLT1	6
32	TRPA1	16	29	PLAT	5	32	PLAU	6
33	PLAT	14	29	PTGFR	5	33	NR1H4	5
34	PLA2G2A	12	29	PPARA	5	34	PARP1	5
34	PPARA	12	35	NR1H4	4	35	PLAT	5
36	PARP1	10	35	LTB4R	4	36	PTGFR	5
37	NR1H4	7	35	PLA2G2A	4	37	CYP2C19	4
37	CYP2C19	7	38	CYP2C19	3	38	PLA2G2A	4
39	KCNK3	6	38	KCNK3	3	39	FABP1	4
39	KCNK9	6	38	KCNK9	3	40	KCNK3	3
41	FABP1	5	38	FABP1	3	41	KCNK9	3
42	GBA	2	42	GBA	2	42	GBA	2

**Table 3:** Docking score of highly enriched pathway targets of inflammation with key bioactives, standard medicines and theirs co-crystallized ligands.

Gene	PDB ID	Bioactives	Docking Score Kcal/mol
TLR4	3UL7	N-(3-chlorophenyl)naphthylcarboxamide	−4.027
		Aspirin *	−4.178
		Indomethacin *	−4.168
		2-acetamido−2-deoxy-beta-d-glucopyranose	−4.797
EGFR	5WB7	N-(3-chlorophenyl)naphthylcarboxamide	−4.123
		Aspirin *	−4.182
		Indomethacin *	−4.707
		2-acetamido-2-deoxy-beta-d-glucopyranose	−4.205
FLT1	3HNG	N-(3-chlorophenyl)naphthylcarboxamide	−8.88
		Aspirin *	−6.794
		Indomethacin *	−5.538
		N-(4-chlorophenyl)-2-[(pyridin-4-ylmethyl)amino]benzamide	−11.044
NOS3	1M9J	N-(3-chlorophenyl)naphthylcarboxamide	−7.236
		Aspirin *	−5.262
		Indomethacin *	−7.451
		Chlorzoxazone	−6.118
NOS2	1NSI	N-(3-chlorophenyl)naphthylcarboxamide	−8.85
		Aspirin *	−6.467
		Indomethacin *	−7.491
		Protoporphyrin Ix Containing Fe	−14.755

Notes: * denotes standard medicine.

## Data Availability

All data are provided in this paper and supplementary materials.

## Supplementary Materials

The following supporting information can be downloaded at: https://www.mdpi.com/article/10.3390/life13040893/s1, File S1: Table S1: Drug-likeness properties of 27 bioactives from methanolic extract of W. extensa. Table S2: Bioactives ranking order in Bioactives-Targets network based on degree value. Table S3: Inflammation related targets enrichment in 10 signaling pathways. Table S4: Details binding interaction of key compounds with HIF-1 signaling pathways genes. Table S5: Different quantum parameters of key compound of WE and standard drugs. File S1: Figure S1: Protein-Protein Interaction using different algorithm. File S1: Figure S2: Gene Ontology (GO) analysis of common targets between bioactives and inflammation. File S2: Compound related gene. File S3: Common genes between SEA and STP. File S4: Inflammatory disease related genes. File S5: Common genes between inflammation related targets and compound related overlapping genes.

## Abbreviations

SEA	Similarity Ensemble Approach
STP	Swiss Target Prediction
OMIM	Online Mendelian Inheritance in Man
MEWE	Methanolic Extract of *Wolfiporia extensa*
HIF	Hypoxia Inducible Factor
MNC	Maximum Neighborhood Component
MCC	Maximal Clique Centrality
KEGG	Kyoto Encyclopedia of Genes and Genomes
FDR	False discovery rate
PPI	Protein-protein interaction
SMILES	Simplified molecular input line entry system
NFkB	Nuclear factor kappa B
EGFR	Epidermal Growth Factor Receptor
TLR4	Toll Like Receptor 4
FLT1	fms related tyrosine kinase 1
NOS2	Nitric Oxide Synthase 2
NOS3	Nitric Oxide Synthase 23
