# Supplementary material for: Bioactive Compounds and Signaling Pathways of Wolfiporia extensa in Suppressing Inflammatory Response by Network Pharmacology
Source: Life (Basel). 2023 Mar 27;13(4):893. doi: 10.3390/life13040893 (PMC10142087; doi:10.3390/life13040893)
Supplement: Supplementary file 1 [file life-13-00893-s001.zip › Supplementary file 1.pdf]

Table S1: Drug-likeness properties of 27 bioactives from methanolic extract of *W.extensa*

Bioactives	MW	HBA	HBD	MlogP	Lipinski's Violations	Bioavailability score
	<500	<10	≤5	≤4.15	≤1	>0.1
N-Cyano-3-oxobutanamide	126.11	3	1	-1.04	0	0.55
2,4-Dimethyl-1,3-cyclopentanedione	126.15	2	0	0.31	0	0.55
3-Hydroxy-2,3-dihydromaltol	144.13	4	2	-1.77	0	0.85
2-Butynol	70.09	1	1	0.6	0	0.55
2-Butenoyl chloride	104.53	1	0	0.9	0	0.55
Propargyl isothiocyanate	97.14	1	0	1.98	0	0.55
3,4-Trimethyl-5-pyrazolone	126.16	2	1	0.3	0	0.55
2-methyl-2-heptenal	126.2	1	0	1.97	0	0.55
Hexylamine	101.19	1	1	1.53	0	0.55
alpha-Hydroxyisobutyric acid cyclic butaneboronate	170.01	3	0	0.69	0	0.55
Galactitol	182.17	6	6	-2.77	1	0.55
Serine	105.09	4	3	-3.91	0	0.55
1-Methoxy-1-octadecene	282.5	1	0	5.02	1	0.55
Myristic acid	228.37	2	1	3.69	0	0.85
Sorbitol	182.17	6	6	-2.77	1	0.55
13-Methylpentadecanoic acid methyl ester	270.45	2	0	4.44	1	0.55
Palmitic acid	256.42	2	1	4.19	1	0.85
Methyl linoleate	294.47	2	0	4.7	1	0.55
Linoleic acid	280.45	2	1	4.47	1	0.85
6-Chloro-1-nitronaphthalene	207.61	2	0	2.5	0	0.55
Hexahydropyrazin-1-propylamine, 4-[2-[3,4-dichlorophenyl]ethyl]-	316.27	3	1	2.68	0	0.55
Thymidine	242.23	5	3	-1.15	0	0.55
Tricaprylin	470.68	6	0	4.66	1	0.55
N4-hydroxycytosine	127.1	3	3	-1.28	0	0.55
(3.beta.)-Ergosta-5,7,22-trien-3-ol	396.65	1	1	6.33	1	0.55

N-(3-chlorophenyl)naphthylcarboxamide	281.74	1	1	4.34	1	0.55
.alpha.-Ergostenol	400.68	1	1	6.54	1	0.55

MW= Molecular weight; HBA= Hydrogen bond acceptor; HBD= Hydrogen bond donor

Table S2: Bioactives ranking order in Bioactives-Targets network based on degree value

Rank	Bioactives	Degree	Rank	Bioactives	Degree
1	N-(3-chlorophenyl)naphthylcarboxamide	31	15	2-Butenoyl chloride	0
2	Myristic acid	10	16	alpha-Hydroxyisobutyric acid cyclic butaneboronate	0
3	Linoleic acid	10	17	Sorbitol	0
4	Palmitic acid	9	18	Galactitol	0
5	Methyl linoleate	8	19	3-Hydroxy-2,3-dihydromaltol	0
6	Serine	2	20	2,4-Dimethyl-1,3-cyclopentanedione	0
7	Hexahydropyrazin-1-propylamine, 4-[2-[3,4-dichlorophenyl]ethyl]-	2	21	2-Butynol	0
8	Tricaprylin	2	22	3,4-Trimethyl-5-pyrazolone	0
9	Propargyl isothiocyanate	1	23	2-methyl-2-heptenal	0
10	Hexylamine	1	24	N-Cyano-3-oxobutanamide	0
11	1-Methoxy-1-octadecene	1	25	6-Chloro-1-nitronaphthalene	0
12	13-Methylpentadecanoic acid methyl ester	1	26	N4-hydroxycytosine	0
13	(3.beta.)-Ergosta-5,7,22- trien-3-ol	1	27	Thymidine	0
14	.alpha.-Ergostenol	1			

Table S3: Inflammation related targets enrichment in 10 signaling pathways

Term	Targets	Q-Value
hsa04020:Calcium signaling pathway	PDGFRB, PTGFR, HRH1, NOS2, NOS3, F2R, PTGER3, CD38, EGFR	3.25E-05
hsa04015:Rap1 signaling pathway	PDGFRB, FLT1, CNR1, F2R, MAPK14, FGF2, EGFR	0.004655
hsa04066:HIF-1 signaling pathway	FLT1, NOS2, NOS3, TLR4, EGFR	0.010617

hsa04151:PI3K-Akt signaling pathway	PDGFRB, FLT1, NOS3, F2R, JAK2, FGF2, TLR4, EGFR	0.017405
hsa05145:Toxoplasmosis	NOS2, ALOX5, JAK2, MAPK14, TLR4	0.021756
hsa05200:Pathways in cancer	PTGER4, PDGFRB, NOS2, F2R, PTGER3, PPARG, FGF2, EGFR	0.041407
hsa04014:Ras signaling pathway	PDGFRB, FLT1, PLA2G2A, PLA2G4A, FGF2, EGFR	0.060278
hsa04810:Regulation of actin cytoskeleton	PDGFRB, F2R, F2, FGF2, EGFR	0.209022
hsa04611:Platelet activation	NOS3, F2R, PLA2G4A, MAPK14	0.258374
hsa04370:VEGF signaling pathway	NOS3, PLA2G4A, MAPK14	0.329057

Table S4: Details binding interaction of key compounds with HIF-1 signaling pathways genes

Gene	Ligand-Protein complex	Binding interaction		
		Hydrogen bond interactions	Hydrophobic Interaction	Others Bond
TLR4	3UL7 - N-(3-chlorophenyl)naphthylcarboxamide	LEU-119 (1)	HIS-148, PRO-145, LEU-119 (3)	-
EGFR	5WB7 - N-(3-chlorophenyl)naphthylcarboxamide	HIS-409 (1)	PHE-45, ARG-29 (2)	-
FLT1	3HNG - N-(3-chlorophenyl)naphthylcarboxamide	ASP-1040, GLU-878, CYS-1039 (3)	VAL-891, LEU-1013, ILE-1038, VAL-841, ALA-859, LYS-861, VAL-892, VAL-909, LEU-882 (10)	LYS-861 (Electrostatic) 2, LYS-1039 (Pi-Sulfur)
NOS3	1M9J - N-(3-chlorophenyl)naphthylcarboxamide	TRP-356 (1)	VAL-336, PHE-353, PRO-334 (3)	-

NOS2	1NSI - N-(3-chlorophenyl)naphthylcarboxamide	CYS-200, ARG-700 (2)	TRP-194, PHE-369, MET-434, TRP-372, CYS-200 (10)	MET-434 (Sulfur) 1

Table S5: Different quantum parameters of key compound of WE and standard drugs

Compound	HOMO (Kcal/mol)	LUMO (Kcal/mol)	Eg (Kcal/mol)	η (Kcal/mol)	S (Kcal/mol)
N-(3-chlorophenyl)naphthyl carboxamide	-0.224	-0.0589	-0.1651	0.0825	12.115
Indomethacin*	-0.2039	-0.0635	-0.1404	0.0702	14.246
Aspirin*	-0.2546	-0.0546	-0.2	0.1	10

* = Standard drugs, Eg = Energy gap, η = Hardness, S = Softness

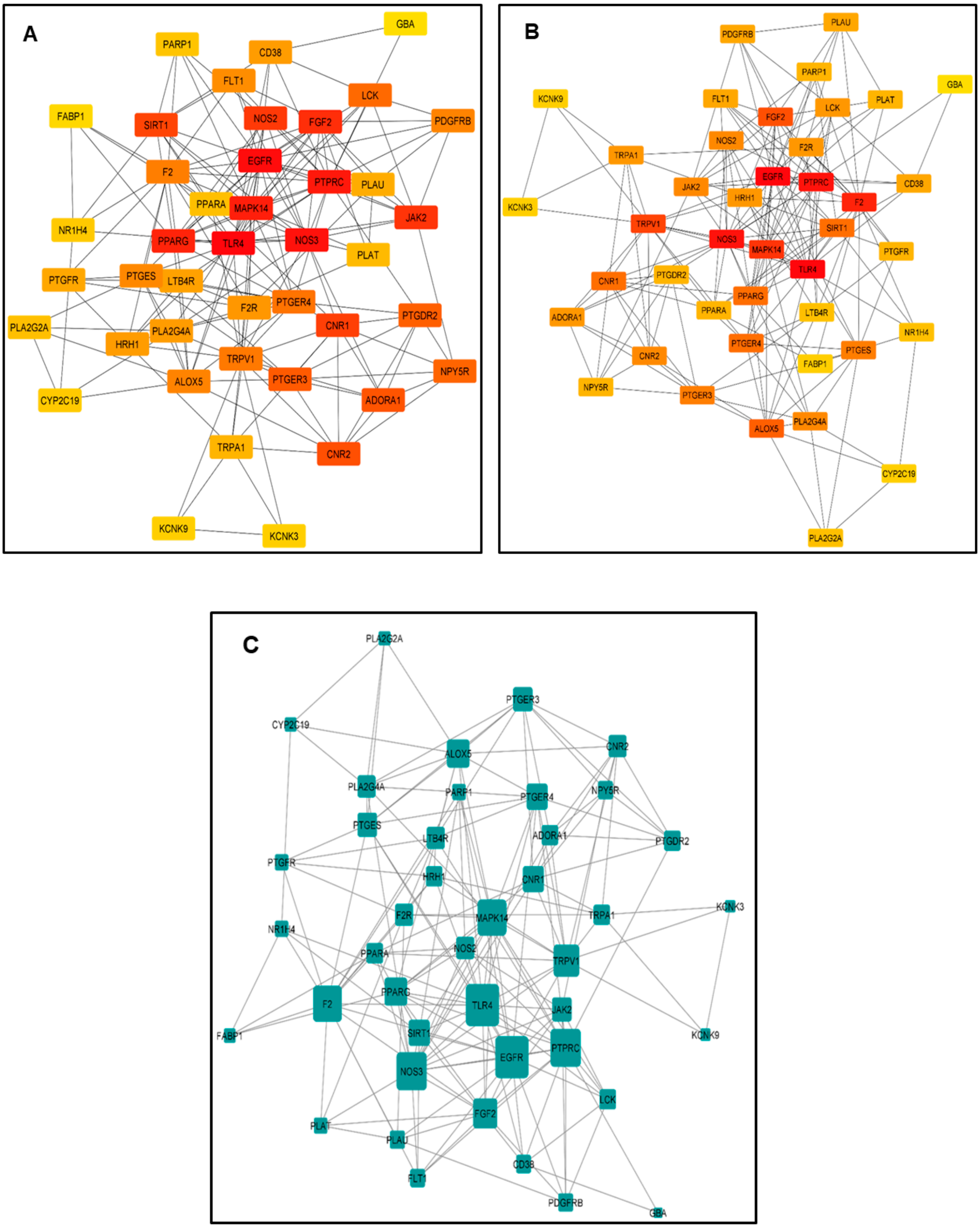

Figure S1: Protein-Protein Interaction using different algorithm; (A) MCC, (B) MNC and (C) Degree

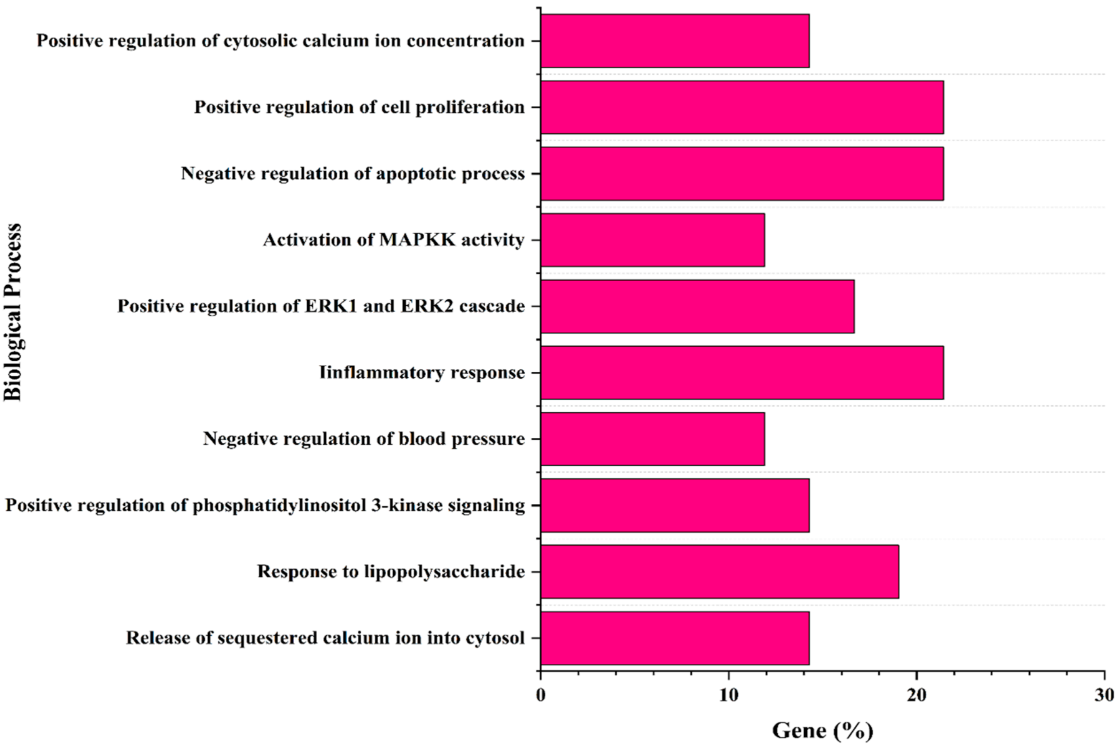
Biological Process
Positive regulation of cytosolic calcium ion concentration
Positive regulation of cell proliferation
Negative regulation of apoptotic process
Activation of MAPKK activity
Positive regulation of ERK1 and ERK2 cascade
Iinflammatory response
Negative regulation of blood pressure
Positive regulation of phosphatidylinositol 3-kinase signaling
Response to lipopolysaccharide
Release of sequestered calcium ion into cytosol
0
10
20
30
Gene (%)

(A)

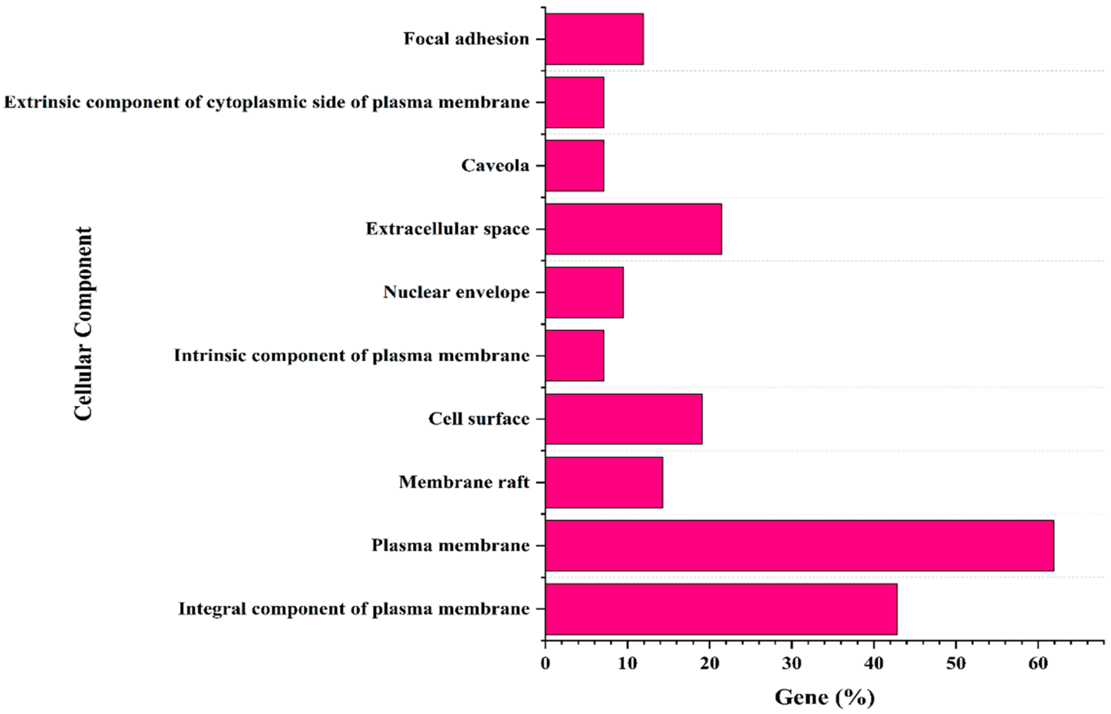
Cellular Component
Focal adhesion
Extrinsic component of cytoplasmic side of plasma membrane
Caveola
Extracellular space
Nuclear envelope
Intrinsic component of plasma membrane
Cell surface
Membrane raft
Plasma membrane
Integral component of plasma membrane
0
10
20
30
40
50
60
Gene (%)

(B)

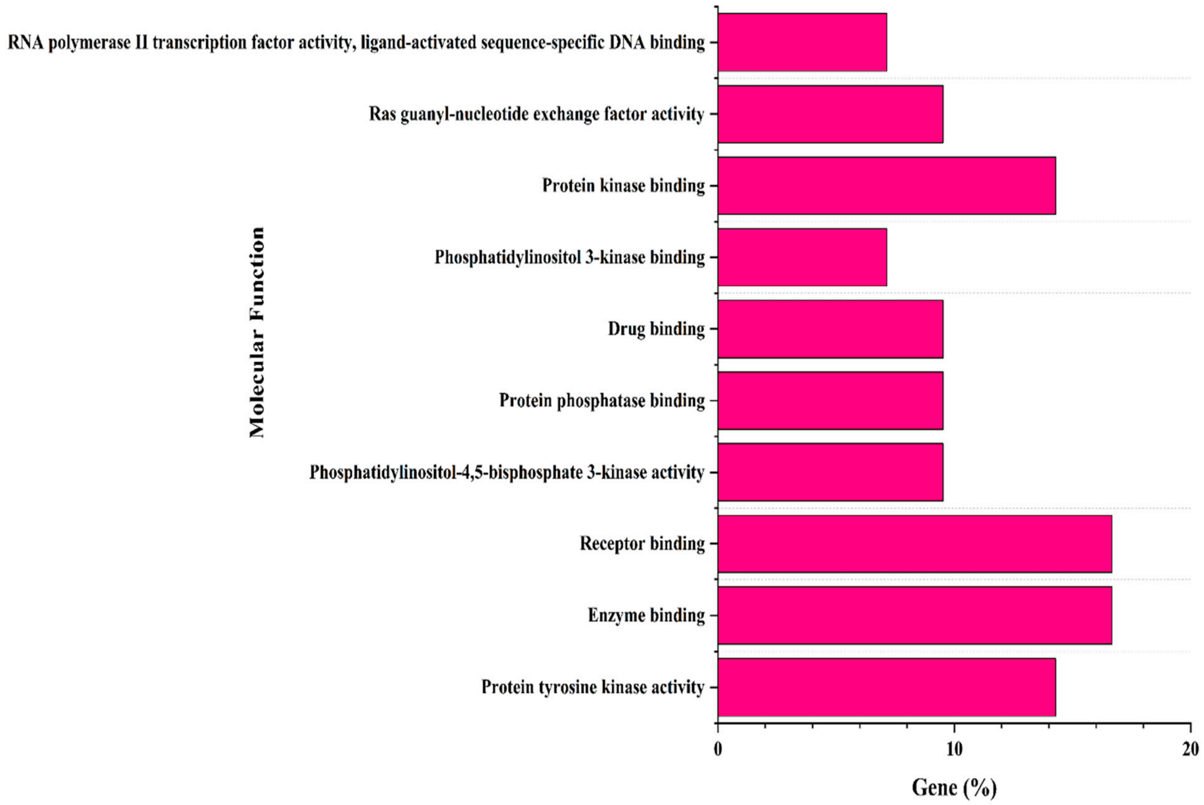

(C)

Figure S2: Gene Ontology (GO) analysis of common targets between bioactives and inflammation; (A) Biological Process (BP), (B) Chemical Contents (CO) and (C) Molecular Function (MF)
