# Supplementary material for: Bioactive Compounds and Signaling Pathways of Wolfiporia extensa in Suppressing Inflammatory Response by Network Pharmacology
Source: Life (Basel). 2023 Mar 27;13(4):893. doi: 10.3390/life13040893 (PMC10142087; doi:10.3390/life13040893)
Supplement: Supplementary file 1 [file life-13-00893-s001.zip › Supplementary file 2.pdf]

<u>Compound related gene:</u>

SEA	STP
608 elements included exclusively in "SEA":	730 elements included exclusively in "STP":
PHF8	CTSK
TRPA1	CTSL
PDE3A	UPP1
ADH1A	FUCA1
ADH1B	P4HA1
ADH7	P4HTM
CES1	EDNRA
CES2	CASP3
ADH1C	CASP6
PAM	CASP7
LAP3	CASP8
CA3	CASP2
CA5A	CHRNA4
CA5B	ADH1A
CA6	AR
DNM1	NR1I3
FDPS	MCL1
GGPS1	ADH1B
GBA	CES1
GNAI1	MAOA
GNAI3	MAOB
GNAO1	MAP2K1
EPHX1	IDO1
LPAR2	CDC25B
LPAR3	DUSP1
PAOX	BCHE
SLC22A1	ACHE
SLC22A2	PTPN22
SLC22A6	PTPRC
SLC22A8	PTPN1
SPHK1	DNMT1
RELA	DNMT3L
TLR8	TOP2A
MGAM	PARP1
RNPEP	FYN
ENPEP	MAPK1
ARG1	CES2
ARG2	CYP19A1
BHMT	TDP2
BHMT2	KDM4B

CTH
DDAH1
SLC1A3
SLC1A2
SLC1A1
GFPT1
GRIA1
GRIA2
GRIA3
GRIA4
GRIK1
GRIK2
GRIK3
GRIK5
GRM6
GRM8
GCLC
GSTK1
KIF11
KYNU
SLC7A5
NOS1
NOS2
NOS3
OTC
PTPRA
SLC15A1
SLC15A2
TH
CNR1
FAAH
FABP3
GPR18
EPHX2
LPAR1
LPAR4
LPAR6
OXER1

P2RY10
PAFAH1B2
KCNK2
ACER2
ATG4B

KDM5B
KDM4A
KDM4C
GSK3B
SIRT3
SIRT2
NOS2
TRPA1
ALOX5
KDM2A
PHF8
CA1
CA4
MGLL
KAT2B
CA2
CYP2A6
CHRM4
CHRNA4
CHRM5
CHRM2
CHRM1
CHRM3
SERPINA6
SHBG
HSD17B3
G6PD
GABBR1
PLK1
CHRNA7
TYMP
PAOX
SLC22A6
CYP11B1
CYP11B2
VDR
NPC1L1
NR1H4
GABRA2
CDC25A
GPBAR1
AKR1B10
POLB
HAO1

BBOX1
CERT1
CYP4F2
CPT2
DAGLA
HSD17B3
POLB
POLL
POLM
ENPP2
FABP4
FFAR4
FOLH1
GABBR1
GABBR2
GABRR1
GABRQ
GPR174
GPR34
GPR35
GPR84
GSR
GSTA1
GSTM1
HAO1
HMGCR
KDM2A
KDM4A
KDM4C
KDM4E
KDM5A
KDM5C
KDM7A
PRKCA
LTB4R
SELL
SELP
SLC25A20
MPEG1
CDC25A
CDC25B
CDC25C
NAAA
NAALAD2

CA7
CA3
CA6
CA12
CA14
CA9
CA13
CA5B
CA5A
TGM2
FABP4
PPARA
FABP3
FABP5
PPARD
UGT2B7
FFAR1
FABP2
ADH1C
KDM5C
TLR9
MMP2
EGFR
MIF
MMP9
PARP3
RGS4
TYR
DAO
GSR
AHCY
HPRT1
ADORA2A
CDA
TK1
PTPRB
PTPN2
ADK
ADA
NOS1
HEXA
HSP90AA1
HEXB
OGA

NOD1
SMPD2
PLA2G1B
PLA2G4A
PLA2G4B
PLA2G5
PLA2G2A
PLA2G2C
PLA2G10
PTGER2
PTGER3
PTGER4
PTGFR
PTGIR
POLH
POLK
ACP1
PPARA
PPARD
PPARG
RARB
S1PR2
S1PR1
S1PR4
S1PR3
S1PR5
SLC6A11
SLCO2A1
SPTLC2
TBXA2R
TBXAS1
THRA
THRB
TLR2
TRPV1
KAT2B
PTPN13
NDUFAB1
APEX1
NDUFAF1
CNR2
POLA1
KAT5
PRKCE
TYMS
PNP
PTGS1
PTGS2
PIM1
AKR1B1
CDK9
CSNK2A1
KDM4E
ESR2
EPHX1
FBP1
PRKACA
AKT1
GDA
GBA
HDAC1
ABCB1
BBOX1
ACE
PIK3CA
RNASEH1
PLAUR
DDAH1
AOC3
BRD4
ACPP
NOS3
ADRA2B
KLKB1
F2
ELANE
PLAU
CTSS
TERT
MAP2
JAK1
JAK2
TYK2
GRM5
KDM5A
ADORA1
PDE7A
ADORA2B

ALOX5	CDK2
LPAR5	GRM2
LY96	PDE4B
NDUFA1	PDE4D
NDUFA2	NAAA
NDUFA3	GRM4
NDUFA4	ADRA1D
NDUFA5	QPCT
NDUFA6	CISD1
NDUFA8	PARP10
NDUFA7	GABRB3
	CAPN1
NDUFA9	NISCH
NDUFA10	F13A1
NDUFA12	HRH3
NDUFA13	HRH4
NDUFB1	GABRB3
NDUFB2	GABRB3
	GABRA2
NDUFB3	MALT1
	CDK5R1
NDUFB5	CDK2
	MAPK8
NDUFB4	MAPK10
NDUFB6	TLR4
NDUFB7	DYRK1A
NDUFB8	HTT
NDUFB9	SLC6A2
NDUFB10	FAAH
NDUFB11	SLC6A4
NDUFC1	SLC6A3
NDUFC2	KDR
NDUFAF2	HMOX1
NDUFAF3	MET
NDUFAF4	MAPK9
NDUFS2	ADH4
NDUFS1	SLC5A7
NDUFS3	SRD5A1
NDUFS4	HDAC8
NDUFS5	DRD4
NDUFS6	IMPDH2
NDUFS7	DRD2
NDUFS8	ALDH1A1
NDUFV1	CYP2C19

NDUFV2
NDUFV3
MT-ND1
MT-ND2
MT-ND3
MT-ND4L
MT-ND4
MT-ND5
MT-ND6
NDUFA4L2
PDCD4
SPTLC1
IARS1
TLR4
TOP2A
CBS
EcR
ALOX12
MGLL
NDUFA11
TOP1
AKR1C4
EPAS1
HNF4A
EIF4E
NFKBIA
KCNMA1
NCOA1
NR1D1
PDE7A
PDE7B
PHLPP2
PIN1
PLCG2
PMM2
USP47
USP7
VCAM1
HTR1A
HTR1B
HTR1D
HTR2A
HTR2C
HTR7
ADH7
SOAT1
TPO
MPO
NR1I2
FABP1
PIN1
AHR
TDO2
NQO2
P2RX7
PTPRF
LIPE
MTNR1A
MMP13
MMP8
SRD5A2
MMP1
PABPC1
MTNR1B
GLI2
GLI1
CTSC
CTSB
IKBKB
NR4A1
ALOX15
ALOX12
PSMB5
HSPA1A
PTK2B
HTR2B
SIGMAR1
DNM1
TAAR1
KDM1A
PNMT
GRIN1
GRIN2A
GRIN1
SLC22A2
SLC47A1
PDPK1
HTR2A

ADRA1A
ADRA1B
ADRA1D
BCL2A1
CACNA1G
CASP4
CASP5
CCR2
CCR3
CYP2D6
DHCR7
DRD1
DRD2
DRD3
DRD5
DRD4
EBPL
HRH1
HRH4
HRH3
KCNJ1
KCNH2
LIPG
L3MBTL1
L3MBTL3
LOXL2
LOXL3
LSS
LOX
ABCC1
NMT1
OPRK1
PRCP
PTGES2
PROKR1
PNMT
PTPRG
PYGL
SLC6A2
SLC6A3
SLC6A4
PHGDH
SIGMAR1
SMYD2

HTR2C
RCOR1
HTR1B
HTR1D
HTR6
SLC18A2
CHRNA3
DPP4
ADRA2A
HTR3A
CHRNB4
CHRNA3
SPHK1
HTR5A
ADRB1
HTR1A
HTR1E
HTR7
HRH1
DRD3
DPP7
ADRA1A
RET
OPRL1
ODC1
PRCP
ADRA2C
CYP2D6
PLA2G2A
LSS
PLA2G1B
KISS1R
LAP3
ANPEP
KCNH2
KCNMA1
FLT3
PLA2G7
PDE5A
BACE1
ECE2
MPI
JAK3
TTR

TPSAB1
UTS2R
CDA
BCHE
POLD1
POLG
GPR17
TK1
TK2
DTYMK
NT5M
OGT
P2RY2
P2RY4
P2RY6

P2RY14
PYGM
RNASE1
RNASE2
TYMP
TYMS
CYP2E1
PPM1A
VEGFA
SERPINA6
CYP17A1
CYP24A1
CYP27B1
CRYAB
DHCR24
FGF2
G6PD
NPC1L1
NR1H3

RORA
SHBG
SREBF2
VDR
HTR1E
NUDT1
ADORA1
ADORA2B

HSD11B1
PYGM
FNTA FNTB
SERPINH1
HSP90AB1
TNKS2
TNKS
METAP2
CBR1
NR3C1
GABRA1
PIK3CA
AURKB
AURKC
AURKA
EPAS1
ALPL
CXCR2
CTRB1
TNNI3K
CHRNA3
GPR84
STK33
PPME1
BRAF
PARP2
PIM2
RBBP9
FLT1
PSEN2
ADORA3
STS
FGFR1
PIP4K2C
CNR1
NR1H3
POLA1
SHH
PYGB
TRPM8
PYGL
ESR1
PTGER2
PTGFR

ADORA3
ABCG2
ABL1
CHRNA1
CHRNB1
CHRND
CHRNG
ACKR3
CHRM1
ACR
ADRB1
ADRB3
AKR1C2
AKR1C3
GFER
ANO1
AOC2
MAOA
MAOB
APOB
GRK2
GRK3
ATF1
ATR
AURKB

AURKA
BACE1
BAP1
BAD
BCR
BIRC5
BDKRB1
BLK
BRAF
BTK
CACNA1H
CA12
CA9
CASP1
CASP3
CASP6
CASR
CTSD

FOLH1
NAALAD2
SCN9A
FDFT1
GGH
MGAM
SI
CYP51A1
GANC
GLA
ACLY
CPA1
FGF2
HMGCR
GAA
SLC1A1
HDAC3
SLC6A1
GABRA3
GABRR1
SLC6A11
SLC6A13
GRIK1
GRIA1
GRIK5
GRM3
GRIA4
GRM8
GRIK2
GRIK3
GRM1
GRM7
GRIA2
GRM6
GABBR2
TRPV1
ACACB
GPR119
CNR2
NAMPT
CHEK2
VCP
PTAFR
MAPK14

CTSV
AGBL2
CPN1
CD38
CDC42
CDK8
CFTR
HSPE1
HSPD1
CLK2
CLK4
CMA1
CYP1A2
CYP2C9
CYP2C19
CYP3A4
CPT1A
CPT1B
CSF1R
CSNK2A1
CTBP2
CTDSP1
CTNNB1
CXCR5
DCUN1D1
DDR1
DDR2
DDX3X
DGAT1
DNMT3A
DNMT3L
DYRK1A
DYRK1B
DYRK3
EGLN1
EGFR
MLLT1
EPHB3
ERCC1
ERCC5
FBP1
PFKFB4
F10
F12

HCRTR2
HCRTR1
MDM4
MDM2
HDAC6
HDAC2
HDAC10
HPGDS
DAGLA
ABHD6
ACACA
CALCRL
RPS6KB1
CPT1A
LYPLA1
LYPLA2
PPARG
PTPN11
BDKRB2
DHFR
OXTR
FASN
ALOX5AP
EPHX2
F2R
FKBP1A
MPEG1
ROCK2
CHEK1
PLG
ERBB2
CSF1R
PLAT
GLS
F10
MMP3
MMP14
MAPT
APP
SMO
TBXAS1
DGAT1
CCR5
HPGD

FABP1
RCE1
FADS1
PTK2B
FEN1
FLT3
FRK
FTO
FYN
CCKBR
GPR139
GRM1
GRM4
GRM5
SLC2A1
HDAC2
HDAC1
HDAC3
HDAC6
HDAC8
EIF4H
ITPR1
JAK2
JUN
CSNK1A1
CSNK1D
CAMK2B
CAMK2D
CAMK2G
KCNJ11
KCNJ3
KCNJ5
KCNJ6
KCNK9
KCNK3
KCNQ2
KIT
KLF5
KLK1
KLK7
PRKD2
RPS6KB1
LCK
LGALS7; LGALS7B
CETP
RXRA
GSTK1
HSD11B2
RXRB
RARG
RXRG
RARB
RARA
RORB
RORA
SLC22A12
SLC16A1
PTGDR2
TBXA2R
CACNA2D1
PTGER4
RBP4
FFAR4
PTGES2
PTGES
CYP26B1
PTGDR
CYP26A1
AKR1C3
PLA2G4A
AGTR1
PTGER3
THRA
THRB
PGR
CMA1
CTSG
PDE4A
KEAP1
BCL2L1
OXER1
MMP16
TP53
NPY5R
GRIN2B
MTTP
APOB
BRD2

LGALS9
ALOX15
MAP4K4
MARK2
MARK3
MATK
MCHR1
MCL1
MITF
MAPK3
MAPK8
MAPK9
MAPK10
MAPK14
MTTP
NCEH1
NCOA3
NFKB1
NLRP3
GRIN2B
NOD2
NOX1
NPY5R
NR1H4
NR1I2
NR2E3
NTRK3
OPRL1
OXGR1
P2RX7
PLA2G2D
PABPC1
PLA2G7
SERPINE1
PARP10
PARP12
PARP14
PARP15
F2R
PARG
PARP1
PARP4
PTGDR2
PDGFB
BRD3
LCK
RAF1
GCGR
MTOR
LIMK2
CRHR1
EIF4H
P2RY1
P2RY2
P2RY14
IDH1
PFKFB3
PDGFRB
CYP24A1
CDC7
CYP27B1
PDE10A
CASR
MAP3K8
GABRA1
LDLR
GABRA5
CLK4
LTA4H
NR0B2
CDC45
GLRA1
ABCC1
SCD
SAE1
ITGAL
HNF4A
AKR1C1
ENPP2
METAP1
GCG
ITGAL
PRKAG1
MME
CYP17A1
OPRD1
SLC6A9
GCK

PDGFRB
PDGFRA

PKN2
PLEC
ACP3
ALPI
PPIA
CTSA
PRNP
PTGES
PTK6
PTPN11
PTPRC
DHODH
RAF1
RCOR1
RHOA
RRM1
ROCK2
RORB
POLR1A
RXFP2
RXFP1
S100B
SLC12A2
SLC6A12
SAE1
UBA2
SCN2A
SCN10A
SENP1
SENP2
SENP6
SENP7
SENP8
SGK2
TMEM97
SHH
SIRT1
SIRT2
SIRT3
SMARCA2
SMARCA4

SLC9A1
ASAH1
EPHB4
SRC
ABL1
YES1
RORC
OPRM1
CD38
KIF11
BRPF1
CALCRL
BCL2
KIT
HSD17B2
CSNK1D
PREP
LTB4R
PTPN6
TOP1
PTGIR
CD81
PRKCH
ACP1
IL6
GLUL
PTGER1
MAPK3
NR3C2
ADAM17
MC4R
MC1R
MC5R
COMT
LRRK2
USP10
USP13
DDX3X
CCR1
CCR4
CCR2
LIMK1
PDE3A
PDE3B

SMN1; SMN2
SMO
SGMS2
SORT1
SOS1
SRC
STK17A
STAT5A
STAT5B
STAT1
STAT3
STK3
MARS1
SNCA

TAAR1
TASP1
TBK1
TERT
TCF7L2
F2
TMPRSS4
PLAT
traP
TRPM2
TRPV4
TAS1R2
TAS1R3
TSHR
TYK2
UBE2I
UCHL1
PLAU
AVPR1A
AVPR2
FLT1
FLT4
KDR
WNT3
XBP1
ERCC4
ABCB11
ATP12A
CA1

MKNK1
NQO1
ABCG2
PDGFRA
PDE9A
CCNB3
NUDT1
PBK
CAMK2D
PRKCQ
NEK1
PRMT3
KNG1
CXCR4
MELK
HTR4
CCND1
CDK1
MAPKAPK2
PRKCD
CSNK1E
CSNK2A2
HASPIN
CDK9
OPRK1
PRMT6
PRMT8
PRMT1
IRAK4
KHK
PRKCA
TTK
AXL
TYRO3
MERTK
PIM3
ABAT
CHUK
SSTR4
PAK1
SSTR2
DYRK2
PAK4
BTK

CA2
GPBAR1
SLC10A2
SLC10A1
ST3GAL1
GC

CCNE1
AKT2
AKT3
CSNK1A1
PKN2
MAPKAPK5
PIK3CD
PIK3CB
PIK3CG
PI4KB
CYP1A2
FPGS
DCK
DGUOK
RNASE1
ANG
RNASE4
DTYMK
PRKCG
PRKCE
TRPV4
PAM
AGTR2
PPM1B
PPP1CC
PPP5C
PPP1CA
LPAR6
LPAR3
LPAR2
LPAR1
LPAR5
LPAR4
CAPN1
MOGAT2
GSTA1
GSTP1
CAPN2
SLC2A1
ADCY1
P2RY12
NR5A1
CCKBR
CDK5

ECE1
MST1R
LYN
GNRHR
CCKAR
TACR1
BAD
GLRA2
PPIA
CACNA1B
NPY2R
NMBR
KCNA1
KCNA4
ADCY5
ERN1
IGF1R
ALK
MMP7
MMP12
EDNRB
ALPG
PLAA
HCAR2
SIRT1
NT5E
PRKDC
HCK
PFKFB4
NAT1
GC
SREBF2
PRKCB
AVPR1A
REN
S1PR2
PDE2A
PDE4C
C5AR1
KCNA5
AVPR2
SQLE
KCNA3
ICMT

HSD17B1
S1PR3
FADS1
PRSS1
BCAT2
SLC8B1
KCNK3
KCNK9
PGGT1B
PIK3CD
C1R
TLR8
CREBBP
NPBWR1
CDK1
RIPK1
GPR55
MEN1
DNMT3A
SSTR3
CCNE2
SLC18A3
PDGFRA
DHCR7
NR1H2
ATP12A
IL6ST
HIF1A
GPR18
BACE2
