# Supplementary material for: Bioactive Compounds and Signaling Pathways of Wolfiporia extensa in Suppressing Inflammatory Response by Network Pharmacology
Source: Life (Basel). 2023 Mar 27;13(4):893. doi: 10.3390/life13040893 (PMC10142087; doi:10.3390/life13040893)
Supplement: Supplementary file 1 [file life-13-00893-s001.zip › Supplementary file 3.pdf]

Common genes between SEA and STP:

284 common elements in "SEA" and "STP":

PHF8
TRPA1
PDE3A
ADH1A
ADH1B
ADH7
CES1
CES2
ADH1C
PAM
LAP3
CA3
CA5A
CA5B
CA6
DNM1
GBA
EPHX1
LPAR2
LPAR3
PAOX
SLC22A2
SLC22A6
SPHK1
TLR8
MGAM
DDAH1
SLC1A1
GRIA1
GRIA2
GRIA4
GRIK1
GRIK2
GRIK3
GRIK5
GRM6
GRM8
GSTK1
KIF11
NOS1
NOS2

NOS3
CNR1
FAAH
FABP3
GPR18
EPHX2
LPAR1
LPAR4
LPAR6
OXER1
BBOX1
DAGLA
HSD17B3
POLB
ENPP2
FABP4
FFAR4
FOLH1
GABBR1
GABRR1
GPR84
GSR
GSTA1
HAO1
HMGCR
KDM2A
KDM4A
KDM4C
KDM4E
KDM5A
KDM5C
PRKCA
LTB4R
MPEG1
CDC25A
CDC25B
NAAA
NAALAD2
PLA2G1B
PLA2G4A
PLA2G2A
PTGER2
PTGER3
PTGER4

PTGFR
PTGIR
ACP1
PPARA
PPARD
PPARG
RARB
S1PR2
S1PR3
SLC6A11
TBXA2R
TBXAS1
THRA
THRB
TRPV1
KAT2B
CNR2
POLA1
PRKCE
ALOX5
LPAR5
TLR4
TOP2A
ALOX12
MGLL
TOP1
EPAS1
HNF4A
KCNMA1
PDE7A
PIN1
HTR1A
HTR1B
HTR1D
HTR2A
HTR2C
HTR7
ADRA1A
ADRA1D
CCR2
CYP2D6
DHCR7
DRD2
DRD3

DRD4
HRH1
HRH4
HRH3
KCNH2
LSS
ABCC1
OPRK1
PRCP
PTGES2
PNMT
PYGL
SLC6A2
SLC6A3
SLC6A4
SIGMAR1
CDA
BCHE
TK1
DTYMK
P2RY2
P2RY14
PYGM
RNASE1
TYMP
TYMS
SERPINA6
CYP17A1
CYP24A1
CYP27B1
FGF2
G6PD
NPC1L1
NR1H3
RORA
SHBG
SREBF2
VDR
HTR1E
NUDT1
ADORA1
ADORA2B
ADORA3
ABCG2

ABL1
CHRM1
ADRB1
AKR1C3
MAOA
MAOB
APOB
AURKB
AURKA
BACE1
BAD
BRAF
BTK
CA12
CA9
CASP3
CASP6
CASR
CD38
CLK4
CMA1
CYP1A2
CYP2C19
CPT1A
CSF1R
CSNK2A1
DDX3X
DGAT1
DNMT3A
DYRK1A
EGFR
FBP1
PFKFB4
F10
FABP1
FADS1
PTK2B
FLT3
FYN
CCKBR
GRM1
GRM4
GRM5
SLC2A1

HDAC2
HDAC1
HDAC3
HDAC6
HDAC8
EIF4H
JAK2
CSNK1A1
CSNK1D
CAMK2D
KCNK9
KCNK3
KIT
RPS6KB1
LCK
ALOX15
MCL1
MAPK3
MAPK8
MAPK9
MAPK10
MAPK14
MTTP
GRIN2B
NPY5R
NR1H4
NR1I2
OPRL1
P2RX7
PABPC1
PLA2G7
PARP10
F2R
PARP1
PTGDR2
PDGFRB
PDGFRA
PKN2
PPIA
PTGES
PTPN11
PTPRC
RAF1
ROCK2

RORB
SHH
SIRT1
SIRT2
SIRT3
SMO
SRC
TAAR1
TERT
F2
PLAT
TRPV4
TYK2
PLAU
AVPR1A
AVPR2
FLT1
KDR
ATP12A
CA1
CA2
GPBAR1
GC
