# Supplementary material for: Bioactive Compounds and Signaling Pathways of Wolfiporia extensa in Suppressing Inflammatory Response by Network Pharmacology
Source: Life (Basel). 2023 Mar 27;13(4):893. doi: 10.3390/life13040893 (PMC10142087; doi:10.3390/life13040893)
Supplement: Supplementary file 1 [file life-13-00893-s001.zip › Supplementary file 4.pdf]

**Inflammatory disease related genes:**

7283 elements included exclusively in within DisGeNeT, OMIM, MALACARDS database

ISG15
TNFRSF4
C1QTNF12
TNFRSF14
TP73
TNFRSF25
TNFRSF9
PIK3CD
MTOR
TNFRSF8
TNFRSF1B
PDPN
CTRC
PLA2G2E
PLA2G2A
PLA2G2F
PINK1
CNR2
RUNX3
MATN1
CCDC28B
LCK
YARS1
TRIM62
ZC3H12A
HPCAL4
MYCL
CAP1
GUCA2B
TIE1
MUTYH
RAD54L
TAL1
MYSM1
JUN
JAK1
IL23R
IL12RB2
WLS
PTGER3

PTGFR
CCN1
GFI1
F3
S1PR1
GSTM1
CSF1
CHIA
PTPN22
NRAS
IGSF3
REG4
HJV
FCGR1A
CTSK
SETDB1
TNFAIP8L2
SELENBP1
PSMB4
RORC
FLG
FLG2
SPRR2A
SPRR2B
S100A9
S100A12
S100A8
IL6R
ADAR
MUC1
GBA
LMNA
TMEM79
CD1D
AIM2
ACKR1
FCER1A
CRP
COPA
NCSTN
CD244
NECTIN4
FCER1G
FCGR2A

FCGR3A
FCGR3B
FCGR2B
DDR2
F5
SELP
SELL
SELE
FASLG
TNFSF4
RC3H1
STX6
LAMC2
HMCN1
PTGS2
PLA2G4A
KCNT2
CFH
CFHR3
CFHR1
PTPRC
NR5A2
ZNF281
ADIPOR1
ADORA1
CHI3L1
CNTN2
IKBKE
IL10
IL24
CD55
CR2
CR1
CD46
LAMB3
TRAF5
SLC30A10
TLR5
PARP1
AGT
EGLN1
NLRP3
LYPD8
PXDN

SLC5A6
MPV17
FNDC4
ALK
NLRC4
EIF2AK2
SIX2
TTC7A
EPCAM
CCDC88A
GKN2
DYSF
REG1A
VAMP8
CD8A
FABP1
STARD7
NEURL3
MAP4K4
IL1R1
IL1RL1
BUB1
MIR4435-2HG
MERTK
IL1A
IL1B
IL36G
IL36A
IL36B
IL36RN
IL1RN
MARCO
PROC
CXCR4
HNMT
NR4A2
GPD2
ACVR1
ITGB6
DPP4
GCG
SCN1A
SCN9A
G6PC2

NFE2L2
NEUROD1
ITGAV
GULP1
STAT1
STAT4
C2orf69
CASP10
CASP8
CTLA4
ICOS
BARD1
CXCR2
CXCR1
SLC11A1
IRS1
CCL20
SP110
NMUR1
ATG16L1
TRPM8
ACKR3
PDCD1
SUMF1
CELIAC8
COPD
IDDM13
ADA
SH2B3
NAMPT
CDK5
CDKN1A
TLR6
CCL26
KLF2
NOD1
PROCR
TXNIP
TNFSF13B
MASP2
CFTR
LILRB1
LIAS
DUSP10

PTGDR2
TREX1
CHRNA4
CHRNB2
UCN3
TNFRSF13C
ADCYAP1
APOA5
CLU
SERPINA3
CCR5
CCR6
CCR7
CCR8
ACKR2
LTB4R
CNR1
CNTF
COL2A1
CD200R1
ADM
CPB2
CRH
CRHR2
CRYAB
MAPK14
CSF2
CST3
CTNNB1
CTSB
CTSG
CX3CR1
CYP1A1
ADRB3
CYP2C19
CYP19A1
CLEC12A
DDT
DEFA1
DEFA3
DEFB4A
DMBT1
AGER
DSG2

AGTR1
EDN1
EFNB1
EGF
EGFR
EGR1
AHR
AHSG
EIF4EBP1
AIF1
ELANE
ELF3
A2M
EPO
AKT1
F2
F2R
F2RL1
F7
F11
ACSL3
ACSL4
FCAR
FGF1
FGF2
FGF7
FGG
FKBP4
NCOA6
FLT1
FLT3LG
ACSL6
FN1
SIRT1
TNFRSF13B
FPR2
IL17RA
ALOX5
ALOX5AP
IL27
ALPL
GAST
PRDX5
GALNS

PYDC1
GATA3
EHF
GCH1
GH1
BIN1
IFNL3
ANG
CXCR3
PLA2G4D
ANGPT1
ANGPT2
GRN
NR3C1
CXCL1
CXCL2
GTF2A1
PADI1
TBX21
GZMA
ANXA1
HGF
NRG1
HIF1A
HMGB1
HMOX1
HP
APCS
HRH1
HSPA4
BIRC5
HSP90AA1
HSPD1
APOA1
APOA4
ICAM1
NLRP10
ENPP7
AQP8
IFNG
APOD
IGF1
APP
FAS

IL1RAP
IL4R
IL6
CXCL8
IL12A
IL12B
IL13
AQP3
IL15
IL17A
IL18
AQP4
IDO1
CXCL10
AQP7
IRF1
IRF7
ITGA1
ITGAL
ITIH2
JAK2
KCNK2
KCNK3
KLK1
KNG1
LCN2
LEP
LIF
LIMK1
ASIC2
LTF
MIR21
MIR217
MIR22
MIR34A
SMAD3
SMAD7
ASIC1
STS
MAZ
MBL2
MEFV
CD99
MIF

NR3C2
MME
MMP1
MMP2
MMP3
MMP9
MMP10
CD200
MPO
CARD17
MT2A
MUC2
MVK
MYD88
SERPINC1
NCAM1
NFKB1
NGF
NOS2
NOS3
NPPB
NPPC
NPY5R
NTF3
NTF4
NTRK2
TNFRSF11B
OPRM1
OXT
SERPINE1
IL20
IL22
F11R
FOXP3
GAL
PCSK1
PCSK2
TLR7
KCNK9
TNFRSF12A
PDGFA
PDGFB
IL23A
PDGFRB

GHRL
PECAM1
SERPINF1
ADA2
PF4
PGF
PGR
ABCB1
ABCB4
PHB
SERPINA1
PIGF
PIK3CG
PLAT
PLAU
PLAUR
PLCB3
PLP1
ACP5
ATP7B
TLR9
TREM1
POMC
PON1
DLL4
PPARA
PPARG
CLN6
PRKAA1
PRKCD
SELENOS
APOM
PRSS3
PRTN3
RETN
PTEN
PTGER4
PTN
PTPN1
SCUBE2
HAMP
PTX3
CXCL16
RELA

RET
TNFRSF17
BDKRB1
BDKRB2
BDNF
S100A7
S100B
SAA1
SERPINB3
SERPINB4
CEACAM1
CCL1
CCL2
CCL3
CCL4
CCL5
CCL11
CCL13
CCL18
CCL19
CCL21
CXCL6
CXCL11
CX3CL1
SDC1
CXCL12
SELPLG
SLC28A3
NOD2
SFTPD
IL25
BMP4
SLC15A1
SLC22A5
RASL11B
SOD1
SPINK1
SPP1
SPRR1B
BPI
STAT3
STC1
BSG
SULT2B1

BST1
TAC1
TACR1
TACR3
TF
TFAM
TFRC
TGFA
TGFB1
TGM2
THBD
THBS1
TIMP1
TIMP4
C1QBP
TLR2
TLR3
TLR4
SERPING1
TMSB4X
TNF
TNFAIP3
TNFAIP6
TNFRSF1A
TP53
TPSAB1
C3
C3AR1
C4A
C4B
TSC2
C5
C5AR1
TYROBP
UCN
SCGB1A1
VCAM1
VEGFA
VEGFB
VEGFC
VIP
TRPV1
VTN
VWF

YWHAG
YWHAH
ZFP36
CACNA1C
DAP3
CALCA
ADIPOR2
TFPI2
TREML2
SCUBE1
SETD7
CAMK2A
MADCAM1
CAMP
CASP1
PLA2G6
CAT
TSLP
NPFF
SOCS1
PDE5A
TNFSF14
TNFSF13
TNFSF12
TNFSF10
TNFRSF18
CCK
APLN
KYNU
TRPA1
TNFSF18
NAT1
LACRT
SOCS3
IL33
SLC28A2
CD3E
EOLA1
IL32
CD83
ASIC3
CD163
ADIPOQ
PLAA

CD28
CYP7B1
NTN1
CD38
PTGES
CXCL14
CD40
CD40LG
CD44
CD47
CD48
CD68
WDR1
TNFSF15
NR1H4
CDH1
PRT133
ACT077
DFN362
CHR093
CHR284
DRM006
BNN003
CHR088
ACT070
DFF030
MYC008
AST005
ATH013
FBR046
ACT192
MST005
INF037
CLT003
BDY004
NNL005
BDY011
BDY006
BDY010
BDY020
BDY012
BDY019
BDY015
BDY005

BDY017
ALL006
ART022
CRH001
PLM129
ULC004
RHM011
FTT001
CYT002
HYP098
PRD008
ALP042
UVT001
PST095
ISC004
RHN004
PNV001
PRN014
PSR002
PRS012
LNG099
CHR684
ATM095
TNS014
DRM053
AGN016
PST011
TYP009
LPD008
PRT036
NNL006
ALL003
VSC011
ORC001
OST012
SLD003
CYS001
ALL029
VSC007
LYM021
HYP066
ADL010
FTT008
SPN225

SKN016
END086
KDN018
LPP008
SYN007
LVR013
GST044
DBT009
TLS001
ALL014
ART140
MLT020
SRC025
PLC002
HYP266
CRN300
CTR002
GT001
GNG013
BRN024
SPN051
PTR001
MYC007
MCR113
PNM007
DPR016
MCR133
MCR130
MCR120
ACT027
PLM036
CHR005
SPN186
TXC005
DFC004
IRN002
PNC044
THR024
BRN071
BCT022
BCH001
UNX003
INF009
BNR002

ALZ034
MCR115
RPD005
HRT032
MNT002
SLP006
IRR002
PSR001
YMN001
HPT021
PRT251
PRS047
GLC003
LPS004
SYS001
HLC007
GLM007
RCK004
CHL123
HYP836
CRB004
SVR001
CLR023
CHR345
48X005
ANR040
CNG034
KRT019
CNJ013
IRD001
CVD001
IGR001
CRD246
PLM037
ENC004
CRH005
STR067
PRD040
CYS018
PST005
MYS005
HPT001
MCL042
ATR087

IMM167
TRM010
RSP003
ESP024
CHR711
EYD002
ART016
PLR008
ENT011
PRS045
ART138
BCL017
INF038
PRS021
BDY007
PRC016
MCL006
HPT016
OTT002
APP008
LVR012
PRS129
PLY011
JVN010
MJR001
FML018
DRR001
BRS047
CLC063
BRN002
ATR011
INT143
HPT023
GRF003
CNT047
LNG032
ENC018
RNL077
PLM134
BRN022
TRD008
MNN013
END044
INT068

SVR004
ART023
HYP595
SCL052
ILT001
PRP027
CHL066
INT007
ENT004
NTR005
PRS040
ACN011
GST053
MCS002
NRR001
CRB039
HMN044
GRN037
ACT075
ALC033
HYP060
DSS032
PLM010
GST106
END047
OST017
TYP008
INF032
HRW001
SCH015
PRN019
NRP001
ADN018
NRT004
ATM011
MSC003
KWS002
CHL067
RTN023
PNC035
AMY004
SCN036
PLM164
KRT002

MYC084
ART021
LTR001
AMY091
BRN056
BRN004
LYM118
DMY004
GST092
EPL164
ANX010
ESP035
HMS001
RCT015
SCK005
MYP004
PCH007
ALL009
DLT002
PRP080
HRP006
CHL147
KRT006
INF071
PRM236
OST002
SCL015
HYP750
RRH023
BNM022
BNM029
INT066
PLP001
HMP009
SQM006
CHR008
ALL010
CHL068
BPL003
SYS005
URM002
MJR022
MJR024
VGT001

BRC012
GST033
URT010
PYL005
ANT039
ANG054
BLP005
FNS001
TRN020
ART106
DVR002
END072
ESN008
DMN002
PRT037
MDD011
VRL010
SLC006
SCL009
THY032
CHL119
HPT073
DRM010
RLP002
END046
URN019
INC002
MSC005
CHR078
APN008
PRC013
OVR082
PLM035
BRN012
BCK006
FDL002
CRT013
HYP014
ACQ007
GST050
SKN015
MLD018
CHR012
ABD014

PLY041
SCH014
THR092
CMP010
SML038
RSC001
LPT014
GST045
ACT071
EXP004
BND020
INT064
URT039
MGR028
MSC007
CRB037
TMP003
MLN008
ALP008
BDY021
OPT009
RTN017
ADN016
HPT003
PST028
CNN005
SPN052
SPL018
ASP006
ATS007
ATS364
BCT007
ATX038
CHR100
KRT009
PNS012
SZR006
CRB172
END033
ALC006
CLL003
OPT006
ACT134
ESP021

SCH060
FBR047
GST023
TND005
HMN014
ALC007
TNS005
EXN002
47X002
PPT005
GLL018
INT079
OVR042
MSC157
STM007
DSS009
PPL022
LYM027
ATR005
SJG008
NTR004
DGN001
CMM005
OVR063
ATX031
RTN020
ILS001
STT001
SQM002
CRN026
ATN005
HYP086
CHG001
HPT019
BLD134
HMC014
CRV039
PLM034
TYP007
PRP019
BNG032
PHR003
ACT068
ANK001

BRR014
EXT034
MLR004
VGN023
NRM001
ATM022
RTN018
THR014
CRD132
SYP003
MNN009
HDC001
ATM099
ECL001
SBC016
INT324
PRT013
PPL052
ANC002
PYD001
ACT249
PLM001
PRT082
IRN001
PRK057
CHL065
ACT076
BCT002
HDR002
MLT161
IGG001
PNN001
DYS073
DNT012
RTN001
PLV003
KLZ004
LSH001
INT323
LCH002
MCR129
DCR003
LYM017
LMB062

HSH003
IRT001
PK3005
DPH001
LPR021
DDN006
CMM004
HYD058
HPT004
SLP005
CRN027
BCT013
FML011
TXP001
CRD119
PNC041
PRC003
HYP069
DDN001
TKY002
VTR013
PLS030
AGG001
CHC001
CNS004
URT001
SPT004
MLN007
DRY001
GLM045
DYS015
GRV001
ORB006
HRP004
TND004
CHR001
RTN016
IGG007
VRL011
PNG002
BLS001
RLP001
HYP080
PLY017

CHR288
ACT084
RNL114
ART006
GLL048
MYL001
NRV007
LNG064
GLL024
PMP014
HYP043
OCC016
CRB210
DFF005
HYP056
CHR417
RDC002
SLP003
HYP076
CND004
LYM133
ACT049
CRN030
SPH001
RSP021
VSC002
HDR003
PPT001
RCT021
FSC004
HNS001
PNB004
PLL012
PLC008
HMN047
ART101
ORB013
ART062
JVN061
IMP005
DWN001
DBT010
VRL005
GLB002

BRS064
NSP012
VNS003
AGN018
KRT001
INF034
INT002
EXR010
GST040
CNC002
PRC012
ALL008
ORL011
ORL015
DBT006
NRB001
ART012
CLN019
SPN035
MTB004
PLM033
OST003
MCR112
LRY019
CRB009
AST034
TMP001
THR015
SYS071
MSL001
NTR042
CMB007
SCK003
CHL004
INT067
PRP030
BHC003
HYP724
CHL122
CRD003
THY029
AYM001
AMY009
HYP025

BRN014
CRY008
CHR066
BLD140
IRR003
TRN034
CLR108
ART074
ASP003
ARC002
SPN119
ALP103
HYP084
ANT006
FLL008
ATM075
MYL069
ACT003
FRZ001
TRN015
RHM001
DSS008
PLY019
OST159
CRV035
LKS001
ERY036
VRL007
PSR023
BCT004
INT070
OPN001
ANT018
BCT021
INS024
ACT042
ACT135
SVR097
PRK039
INT030
TLN003
PRM329
LKM002
END040

VTL004
ANR048
LBY004
RTN022
THR004
MYL005
ORB008
PRD004
SYM002
MCK029
PRM108
CYS005
VRC005
EPS039
ACT055
NPH012
PRT026
TRC008
MST009
TRP002
BRD025
OBS082
EPD006
PRD013
OPT010
PLG002
CHL028
SNS001
SRC014
GSG001
GLL022
BRN108
PTS002
KPS004
MNC007
MCN007
CHL014
PNC108
SCN007
CRT016
PLR007
KHN001
MXL015
HYD006

EXS001
ADL002
CNT035
ALP009
HMC039
VLV008
THL005
ORL013
ATT013
INS002
THR016
PRS042
EXD008
KDN017
MST019
PYD002
OCL069
IFP003
MDS022
LWC001
HNT016
MTR018
PSY004
HMP002
PSR021
ASP007
BRN038
PRT038
HST010
PRN023
ERY004
NRT001
DWL002
DCR004
ATM024
MND021
GNG012
MNN043
INT395
PLY014
KRT008
CRB086
VCC001
HYD002

ACT017
RHM028
HDN002
ERY003
SQM013
CGH001
MVL001
PRV004
CRN024
PNM013
PLM012
TTN003
CHN059
RTR011
ASB001
VTL009
PNC129
CTN007
PLR022
END057
PRS038
LYM009
OTT001
NRS005
CYT005
PLM101
CHR466
PRG009
PNM006
LPT001
ANG015
OVR094
MST023
VTR007
CLC006
MYL057
HRT012
IDP011
TRC023
RHB024
VRN004
GLC106
TRM003
EPD016

BLR001
PMP001
LYM143
JPN002
ILC002
FNG016
CTN003
CHR431
OBS001
INT394
HRD202
CHL056
ALL012
MYL009
RBL001
HML002
TCL028
LKM005
GNG003
PRC001
INT040
HNC001
IMM106
ATS010
CRY001
MLD001
FML021
VRL012
LYM014
MMP001
HMP001
CLR109
RBS001
PSR017
SYS003
DCB001
CRD016
FCL012
GCH001
NWB001
PNC034
LST001
ELS001
LKM062

MBM001
GLM040
OST115
PRP002
PPL061
CRN017
PYR004
PST059
PRM038
ACT029
LCL006
PSR018
WSK001
LYM002
SCR001
ERD001
BLD062
BRG013
OCL025
CHR048
PLY018
MYS003
THY030
CHR708
MYP006
OST062
ISC015
OCL006
INF023
SPS204
CLS016
CHK001
MTH009
ART004
CHR710
CLL015
MMB011
GLC092
VSC003
PRP016
HMR005
KLD004
PSR028
PSR032

RTN024
RHM035
CGN006
LYM129
VLV010
FBR017
MCR191
TCL003
CRT015
PLY150
ATM104
PRS037
PRL003
TRG019
ACT008
ORF044
MTR014
CHR098
HMR023
AMB001
OVR049
ATN004
HYP074
PNM008
MTR002
MCR088
ART115
RTN008
SHW001
ATX030
CRS001
VNT034
HML018
SDD001
BLL006
CTN015
MLK006
BSL007
HLX001
VGN019
MCR225
TXC011
BNC003
NNN032

SKN019
GLL020
ANL018
KRT007
MLG169
BNM018
ART002
NRD100
VSC058
CMP008
ASB003
FNG017
SML019
EPC002
HST011
FRN006
CRB090
MTR003
SPP008
DVR006
CRD137
AVN001
LYM042
DNT006
SHR001
BLR008
SCH002
CHR070
GRN017
TRG002
PST008
PRP017
INT065
NPH009
SHG001
NNS002
MCL003
MLG079
TRC020
KRN002
PHC003
ADR040
SLM003
LMB024

HYP781
LKM070
ACT200
LNG108
CRN028
RSP019
XLN206
ERY066
MLN069
PST092
DRM011
FCL014
PLY001
PPL021
GLC008
HYP730
EXF001
CRN074
LKM061
CRY005
ADL017
GNT006
SYR001
PNP001
CRV002
CRV045
ATP013
HYP061
CNJ007
ACT058
PSD007
DNG005
BLY001
LKC009
NTR018
RTN003
GLS007
ORL005
STV007
FBR054
TRY001
BRY005
FML035
CMP080

FBR011
DRR009
THR123
SXL003
CRP001
ADL030
MCR141
ING001
STR020
MCH006
3MT016
PNM001
ADR007
ESN005
CHM005
ACT105
FLL037
IMM224
ACT073
BRK012
DRM007
MYC006
MLT180
GRN033
CRD223
URN010
BRN026
YLL002
ART017
WST005
PLS007
LMB050
LPT006
SRP002
ANP001
CHR087
UND004
CLN015
DNT047
IMM219
INT075
CNS002
HYP458
EPD015

LYM033
INT017
HYP068
BLL003
WLL004
CCT002
RTR008
PRM005
PYG006
OST016
APH001
BRS102
SPP010
TST044
LYM022
VLV042
INV006
HML001
ARR042
HMN021
PRM011
HYP005
LNG031
CLL021
PLR001
HYP114
TRN018
SPR034
GNG011
CMP090
INT071
MDD010
ANX004
MCN001
APP009
ART005
FLR002
HYP457
ENC067
MTB008
DBT084
PDT043
TRC003
HNT019

TBR001
WLL006
IMM001
ESP023
AMN001
CRB197
RFL001
URT004
STN011
TBR011
BRN028
HRP011
ESS003
PLV020
MLK004
HRP009
SCH012
IDP048
HRM001
ECT026
MNN017
PHM001
EPG003
CTS002
THR001
ANT023
NCR007
PRC002
HPT007
HMP005
APH002
CYS017
FCS012
INT029
MDD018
LMY002
INF068
KLB003
NPH010
AMN003
INF152
HYP006
SPN369
NVS001

MYL006
HYP088
PRT019
NNR004
TRM002
EPD018
DNT008
ATM006
CNT028
ATM101
PFF001
SCL011
ANT080
MYL031
ACT011
TTT001
TRT003
RYN005
ADN027
PRP036
PNC106
GLM044
LPR001
MVM001
INT304
LKP003
CHR576
BLD044
RSP006
EPD005
THY109
VLV047
ART018
CST005
MCK007
MMM001
LKM071
SPN019
SBR004
CRD001
HGH043
MCR010
DBT005
RST023

AZS001
FCL013
STT002
PNT038
PLR005
PMP006
ORL004
SCR015
TST015
PPL019
PLM006
SMN007
NRF002
VLV011
PRM008
ACR007
CWP001
TND006
ATN021
ATN013
NSP002
URL001
HRD214
RTN014
STR033
OST001
CRY024
HTC003
IMM162
ADT003
INT046
ART067
ATS347
MRN002
URT049
INF077
CNT005
DMN023
ONC002
ACT119
GLY013
MYC087
RNL015
HRD002

FRN011
SPN027
DNG002
INS001
DBT002
GST009
GNT003
CNR004
VRC001
IMM179
CYC010
NTH001
ATM113
FML052
OBS002
CRT017
ASC010
GTR002
SCN006
CRB088
HMN032
DMN026
SPS019
OPT053
LCT002
PRM020
CRY004
CHR086
DBT004
PPL049
PLC005
CRP002
FRN020
TBR025
CLS010
HMG005
MTR088
ADP010
MLT035
MRG003
DMY001
TXC001
PRM039
INT051

LYS002
QDR001
TCL023
GRW007
SPS057
BNG093
MYC015
THY107
THY023
ANV001
LKN025
RNL011
GNT167
PLS011
SDD008
ACN019
DBT081
INV001
ADN002
INC003
VRL003
ADN067
PRN021
MSN001
CRB048
PRS034
MLR002
ATR057
NDL024
DRM054
VRS002
SPN354
CHL159
AST006
OSG001
ANT024
MGR001
PNC059
ICH004
CNT016
VNZ002
CHR038
LNR006
ACR056

CHL075
MJD001
LPD009
AGM001
MCR011
SYS043
TNC002
HMP030
SCR039
MYL013
WRN001
SNL007
ACT167
CMP002
CLR030
MKL001
ESN006
HMF011
HMF004
RTN021
THR005
NRM005
ARG004
FML116
PLY020
MYC019
PRS063
ANM038
ABD017
GST037
TNG001
HPT067
HYP083
SNS003
SBC007
MTL005
TLR001
SMP003
MTR010
HTL001
CHR013
DRM040
ECT041
FBR003

FTL070
MGC001
NPH091
CHN012
PRT018
GNR003
RPD001
OST167
MCR018
CRY014
BRS051
RYS001
OCL020
PNM005
TBR006
ADS004
VSC018
NJM001
XNT001
GNR004
LMY004
BCK003
OCL051
SBS003
PLM031
BLD036
ALG001
HND002
TRC005
BCL002
RCS002
BRN018
DST006
CNT046
CHR072
MLT113
MST004
AND014
GRN009
RNV001
BLS002
ORB003
MLG084
HNT011

SCB001
VRN001
TNG007
CHR003
STC004
BTT014
ACT094
PDT027
EXT033
LRY004
NLD001
EPD009
SKN006
SLP001
MNN002
HYP081
ACT004
RDT013
BLR002
HTT002
BCT006
CNG410
BLR006
FBR086
APN006
SVR005
MLK003
ANG020
ADR022
CRN035
INF158
INF160
IMM003
CRN019
ACS001
NRL005
DSC004
SYS034
SRF006
LNG095
RFR003
YSY001
HMP018
DNG003

OPS001
VLL003
PGM001
ALP061
ATY001
LYM051
CD4008
NNT021
HYP008
LPM005
MXD005
ABD010
CRV030
PNC001
SDR002
TRT010
HMP007
NRD033
ATX019
QNQ002
PRP009
ATN024
BLC007
PLY118
ACT036
DCT002
PCH010
DRG024
LYM010
PRK001
NCR004
END021
AVS003
RSP007
HMN010
EPD076
MCP040
AND005
PTN014
ERY029
CRN006
ALC028
SVR003
OTS001

ELP001
GRN003
GST039
IGN003
ECT006
CCC001
IMM225
OMN001
ALV001
OST011
PRM226
DSC009
NDL003
IMM136
WLM013
SPN362
HRT011
CNJ012
ALX002
INT010
HPT014
SCK001
SML001
GRD001
VGN020
CVR002
CRN025
BLR013
MJR007
FSC002
MLT157
SFT003
LYM043
OSS001
ANG002
CPL003
FLR001
RST001
BSD001
ASP008
HRS035
HYP272
APL001
PLM175

PLN005
SBC001
ACH005
GNT019
CHL152
INT146
CCC002
SCL018
ANL022
FBR031
FCL005
HPT022
CHN011
MYM013
BRS044
DMN031
DNG001
HPT015
MYC014
PPL001
OVR029
ETH009
INF002
LYM113
CHR091
NRM002
PPL002
TCK001
SKN005
RTT002
NRS003
ANR004
TRC022
MTR012
OVR046
PLC007
FBR009
ANS011
PTH003
ADN011
NRN002
HRT015
KHL003
ACT012

PRP029
WHP001
ATN022
KRT013
TRC031
AST056
MYL080
HMP022
ECT005
MNN020
STM006
ACL001
DYS032
CLN003
SPR010
IDP049
AST052
ECH003
SLV003
DDN004
PRS036
ANG005
CCN002
LTX001
CYS003
PCK003
FBR064
BCT020
MTY003
STN012
MDL005
ATM045
HYP024
FCH002
DGR001
PST010
NMN002
LRY017
FBR019
ASP026
ASC001
BLL004
BLP006
MYF002

BRG001
GLY060
LYM035
SCR008
MLL001
TRN055
MSC089
PYL004
NCR015
PST021
LRY018
BPL002
ATN011
FBR012
LCH016
HMP012
PLT004
GRM004
SCL048
BRB001
CHR063
ANT002
HPT009
NSS002
3HY005
CHR661
HMC002
MLT009
ALC009
CRC021
AGR019
ACT228
NPH003
MYC013
TBC004
VNT002
HNT002
CRY003
NRM004
ULC001
DSS001
HYP806
ALP004
MYC033

ABD002
DRG002
CNN003
OST009
PRT029
END084
TST014
MLT001
RHM036
ORL007
SLL001
SYN036
HRN029
MYF001
LMB006
SPT005
PRT122
LKM063
MRF001
FRM003
ATN002
FNC027
WLS001
HMG002
QFV001
PRM013
UTR058
MCR020
VSC019
CHR097
TXC007
BRK010
BCP001
MSN002
STR077
THR012
INH020
GST027
ORL012
SMK004
LNG039
LKD001
DRS001
CNG171

SCH003
ATM023
CHR607
CHR073
MRK001
GST019
RHB003
PYG003
KWS001
ACT022
BLD063
RRT020
ACR001
INF067
CYS009
CHR037
THR013
ORB010
SPP011
CRN037
HYP072
ADP007
CHL013
IGG009
STT009
SPP003
PRG014
LPS002
SRC027
ZKF001
OCH001
TRC088
DYS021
TRC026
HYP040
XNT003
PRP034
SRC023
PYR010
FCT005
CRN020
THY020
CRN036
CLC001

MCR013
HRT035
CHD004
VTR003
PRN035
ENT008
RSP027
CFF003
TBR008
SPR120
OPT003
NNT024
DQR001
CRN243
SHR059
CLC004
HMR002
DYS030
INT253
GST012
MNN021
MNN005
HYP020
IGG008
EPD070
IDP081
XRD010
LRY044
HPR003
RSD004
ORT004
RHM014
ECT062
MSC190
AMP013
SVR002
END011
ENC005
PNM010
NRN001
CRT084
PHY002
FNG006
INF058

OCL022
LMY014
ACR005
PLC009
VXS001
PRD041
EXC002
CRC006
ACT035
MCC002
PRN039
PYR009
ACT162
SYP001
KND001
BRS099
CHN004
ANG061
HYP063
CRN270
CVR010
OST160
STR103
URM005
PST106
INT054
LYM024
ATM112
TXC002
ALP101
DFF003
NRF007
ANR007
EXT007
ASC003
LGN005
CYN002
PRR001
INF063
ASP004
EPD061
BP1002
CNV018
MNR012

OPT002
SML011
CYS039
ESN015
JJN008
LCR014
HYP193
SPN020
ACN002
CMP064
DYS154
ART010
SPP007
AMB002
EYL002
ATM089
EVN001
ENC008
BRN009
CYS008
BLD131
HRP025
CVR006
INC021
MMB001
TRP004
HMR013
HYP572
CTS005
BRN101
PRX003
MSC165
CLF027
ANG046
PRT058
ADN012
CHL039
ORB009
ORB020
FML001
PHC015
FML063
GLS018
PST029

BRY001
SPN041
CNG069
CHR178
MYC005
STN013
HVY002
KLD003
CTN020
ALC005
OTM001
AFR002
EGG001
HYP077
INF093
PRS130
FNC043
PRS136
CLN045
BRL010
CNT007
RCT020
MYC017
LPD014
RTC012
PLM016
HYP017
QLT008
HPT046
ESP020
TRC012
LNT001
ACN010
TCD001
NWC001
CTR027
CRT004
TRC037
EPD077
INF072
MLT092
PST046
PST062
PYM002

NDL008
HRN026
C1N001
KRT047
GLB001
BCL004
CHR074
PRR013
SBD001
CRV031
MLT006
UPP004
CNG033
UVP001
MYS079
CRT056
AST055
PNC025
ACT201
NRF024
HYD005
MJR006
MJR003
CRB016
BRN015
NNT033
PLM064
NRN004
T2H001
EXT006
RSP002
PSD087
CHR031
PGT001
STR008
IDP091
PYM001
MPL001
PHN003
LNG028
EST004
MSC152
ATR054
ATN014

OVR096
RHN002
TRG001
RGN005
LNG109
CMM008
OBS004
CNT060
CHR036
FRN014
MCL027
MYS033
BNG095
ESP018
ACT030
MRC003
HRD022
HNT004
FML037
CHL045
DPH024
BLN010
PRC031
OLG001
PRQ002
WHP002
HYP733
INT078
SPR066
CHD001
PRD039
LYM007
LTN029
LRG001
ADL027
ATR024
PLN007
VST001
INF170
INF064
RTR017
SCL005
TNG009
CRB033

ORP003
NPH018
PRT010
TXC010
HLS001
LCT001
BLN008
CHR040
CHL070
INF019
STR022
SKN022
SPH007
BLP003
VTM002
ACT088
MTC005
PST053
MLR024
MND023
FML347
NNN030
MRS001
PLS006
OVR059
SLN001
CNT015
LTN001
HYP828
PST054
MGL007
CRN288
INT358
CRB008
SCT002
BRT043
MNC006
PRD006
MYM001
PHY008
APP010
NNT012
LPD004
ESP025

GST049
PRP005
MSL004
HYP838
DFF031
NRG003
GCH015
GRN062
MRR003
URT037
NRC002
DRR003
PGM029
ORN001
EYL005
PMS001
ECZ002
NM001
SBC012
QLT002
MCP033
ETN001
LYM015
HRD008
EPL050
PRP083
PRM024
NRL016
LYN001
GLC001
CHR060
ESN002
LPG001
END075
ERY001
BNG036
ACT066
HRS011
ASY002
GRN007
GLB021
HMC009
CHR285
IDP074

BHR001
VSL002
DFF006
HML033
PLY105
ABD004
INT099
AGR002
PLS009
ATM021
RYN001
SBC019
RCR001
PRF002
CRT072
VSM001
HLL004
LCR004
BRC010
HYP611
SPS007
NRN021
GRM010
HRD217
BRT054
CNG499
SGN002
ATM052
HYP784
ACT047
MGS001
SHP001
MST020
XRD029
PNM002
LNS003
NNT058
CRB031
LTT001
CRK001
XRP001
ACT069
SCN069
ACR006

SKN020
MTC133
HDG002
EBL001
ENT005
CYT004
SCR003
SPP005
HYP726
SBC003
PRN007
TNS004
PTL009
FML068
CRV069
TNG004
APL002
ANG068
BLD051
CLN044
HPT008
FTZ002
SLP002
DST016
GLL008
MMM006
LWG006
ACT038
DXR001
FBR089
NRG002
MLG069
MCN017
VNW001
ACT079
ANG004
MTP001
NNN006
CMP040
ACT032
FML012
PRT011
BKV001
CRR012

SPS003
PLZ001
KRN004
CRN322
ALK013
SYR010
KRT005
MYS004
HYP035
MTC069
TBL025
ATM096
GRV008
RRL003
EWN003
THY111
END014
THY125
PST047
MCP006
DRF001
LRN003
IMM028
PRG090
LKR002
VTM027
STH001
DNT014
CKT002
NCR008
VND001
GDP001
LGN006
SKN067
TNP001
SNG003
PRT107
SCL013
IND016
END071
XNT002
VSL013
BLD124
PLY179

END041
PLY100
SHL001
BLN009
NDL007
PLM014
ENT001
MYC026
NMN015
PRM004
EHL001
MCR004
FML187
IDP069
TNG002
ADN001
ERY008
TYM001
SCH036
END062
CNT057
LYM019
GNR002
OVR106
PCD001
PRP007
PRC049
FXF002
OCC008
FLT006
CRT002
LYS012
FRG001
ACC008
LKR001
CMP052
ACT164
TYP031
CHL137
T2L001
MCK004
ANG011
ATR010
MGR003

PRS115
AGG012
SPL006
PRG011
LNT004
EPT021
IDP070
NMN014
KRT071
KRB001
FTL006
ICH001
FNC042
OPD001
GND004
MYP120
GNG009
CND006
HYP265
PLM017
PTT006
ORL003
MTN002
WTH001
KRN001
BRS111
ANH002
INV005
STR019
LYM126
CRN048
C3G002
OPT066
LGG001
HMN048
MTR001
ACD003
RFR010
NNB001
TCL001
ENP001
ATM020
CLF001
ALM001

EPD062
NPH100
ATN012
HYP873
HRD037
MTC058
IMM079
PRD003
CMP009
PRG013
CRS005
CRT085
LCH001
FND002
INH030
NCR014
SBC035
VSC052
SCR011
PLY010
SCN059
LGP001
KDN013
PRG047
SPL008
FTL001
WHT017
PHG002
AVD001
CHL061
LYM052
IDP041
GLC086
BDD001
MNN042
GLS008
SWT003
RDN001
LPD001
GRN006
PRT031
DCR002
CRY007
ACT037

INF055
PLY188
CNG064
THY121
SPN046
OPD006
PLS002
CHR675
MYC012
PTT046
HMM002
IMM196
MYP095
KRT028
GRN064
HYP692
HPT084
ORB005
ORB004
LYM004
SPL007
CSP005
HYP082
ACT159
CLC008
PGT007
MLR003
BLD054
PHY001
MDD005
ATR003
ACT093
BRT059
MLT163
HYP276
NRF028
HST017
NST002
RHM021
PRM002
WRN003
SCL003
ATS308
INT059

PTY003
CRB059
DRG026
CRB079
PRG042
HMR003
ERY069
AML029
CHL079
BLR004
MYS001
GST105
UMB002
SCH085
PNS018
TBR026
MLN003
PNL011
NCR001
MNN024
DBT008
CRD005
PRM324
PRL012
HYD019
SPP012
ZNC008
HRM005
MNN034
ABD009
RTN004
JNC001
ALP048
SSC001
LCH008
HYP264
LRY007
PRN011
SCL056
CLC061
BLC009
EPD072
PHT013
ANG028

PRM206
PHS029
ANG029
DRR020
DRR013
OLM002
ACN018
TBL032
LVR030
CNG414
BLC011
TRN029
WSK003
HRT039
LTN015
GLY111
JNC002
KND005
CDQ001
ACT081
CHR057
PRT096
MCN008
DYS101
GST051
ERY017
VTM033
RCT017
IMM154
END016
ALC010
CRT008
ALD013
EF001
SPN185
HMG029
RTN041
ALV002
SMT004
MNT001
CLD011
CTS003
BRW006
OPH003

ART068
CRB045
PGM003
BNG030
CLL010
PRS142
LSS003
ACT072
HMM003
SPP001
PRC005
PRM022
ACR008
DFF035
THR100
TTH006
INT072
WRT003
JVN047
GRN021
ONC007
PRL008
TRC097
ASP030
EHR002
OLG003
PLS032
SPR162
MST024
FNC044
OCL004
PRL001
HYP643
INT221
CRB189
IMM228
EHL071
BLN004
FML026
ACT026
ICH011
ERB002
PGT003
LYM116

CHR071
VLV044
IMM174
BRS112
TTH008
MYC080
TMR018
IMM222
HYP087
JVN019
ATM012
MNN016
FCL011
LCR008
HNM002
OSM001
RTN177
GRG001
ESP027
PHC001
SPR009
CRT024
INF186
CRY035
TRT017
CRC039
WST001
TRC109
THY128
PRM003
OVR109
PRC008
BBS001
PTT037
GLN006
RNL007
LCR013
CHL131
MNN032
MSN004
FSS001
PTY002
RDT001
PRM331

PRG071
PSD006
NPH005
EMP002
KMR001
FLT011
LYM040
TRN014
CNG021
EMY001
RLP003
ANL011
PLM180
BLD033
UVL010
HR4001
MNK001
MNT252
BLD170
CCH001
TCK004
HMT002
XLN249
IGG011
SWN001
HYP001
CHR099
VLL006
STT007
EST003
KRT014
MTH008
THR082
PLM029
DBT007
DDN027
INT060
TFR002
MYT002
MCP001
NNT004
PYR016
RNG015
CHR085

SND001
DDN011
ATM100
ASP038
INN002
PRG008
DFF036
CTN019
GDS001
ANL014
EXT056
NSL004
MDS018
VRL008
GST090
CNG012
LYS001
PRN033
PRS053
CSY001
MJR008
SPS150
MJR023
IMM072
AMY027
MJR004
PRM092
SPR126
ANG018
PRM018
ART141
PRP003
INT303
ALT003
EXT022
CRV038
EPL003
IMP004
STS002
GRN042
CMB039
LNR005
ANR009
PSD012

LMR001
CRN004
FCL003
GRN036
IDP064
PHS025
ZNC006
ECT109
DRG025
CRB005
PST103
CTN034
ART110
SYS004
IRR001
GLC009
ALB002
SWL001
PRM327
ECT108
STL001
BLP001
HRP037
END080
ACT059
ART008
PRR002
LMT001
LFT001
PRG043
MCR017
PNG001
STR002
RGH001
BRR002
ERY023
DRG017
OST028
DRG003
HST016
IGG014
TMP019
CYT018
SHR029

SWT002
THY025
PRC050
MLG049
HDR004
CYS004
OVR002
BNL002
HRT006
SCL057
PLY180
HYP058
OBS037
BTN004
ACN001
BTT017
GRN002
TNC003
MYC016
ATM060
SML008
GM1007
UNV001
PLM085
MLG042
LYM006
CHR035
CHR276
PDT025
PRN026
LPD012
ASC009
HMX003
RBF001
PSR025
PHT002
LYM011
GST071
DRM009
GGR001
INF021
BRD005
GYN001
ACT067

OCL010
MLG002
IGG013
ESN013
GNT001
MLT074
TBL003
CRT009
ACR097
WLF001
MYC058
INT016
BLD130
NRV004
BTN002
EMB004
ACT040
CRM001
URC005
OLG020
ESN016
LPM012
TNB001
NVD003
BCT005
PSR016
LWS001
MYX004
TNS007
ALP097
BCL014
INF013
RHN003
URN003
PRC019
MCK005
SHR107
OVR017
PLM005
OCL008
MLT134
PRF005
LBR038
GLC098

CRB196
INF086
OST015
CHR053
MRP001
FBR002
PRN009
PPT002
PLY012
GTT002
MNG007
ADN064
ACH022
ALL027
PTT014
SCN001
PRS030
THY022
ECT004
SYN005
CHR627
PRN049
BRD002
ACT062
CYS010
TCL027
ERS002
TCL024
PRL021
CYN003
BNG077
HYP263
FML117
PWS001
HYP768
GRN051
TMR017
IMM241
NPH027
EPS001
WST004
DRC001
EPD081
LCL022

JVN046
CD4003
LSS001
URG001
PLM022
FNG004
DWR001
BRN032
MND031
KSH004
TRC115
MLT008
LVD003
SBC011
JVN003
PMP002
PLM198
ASR001
AMR003
UND007
SCN068
ASH001
PRG139
CLP005
EST011
ERY010
PST030
TCL008
FRN028
FLL027
GLS004
APR006
PSD078
FLL019
LTH001
SKL001
VGN017
TNC001
ETH004
MDL002
PNC013
CLR003
GST014
STP004

PLY023
GRY002
ANN011
PNT009
AML002
SRS001
THY122
MLR009
CHR077
MTS001
HRD011
HYP016
MLR001
MLG077
MYL003
CTN013
BNS003
FML053
PRS050
PTN009
STR035
SPS090
ACT150
CHR686
FCT007
ATM016
SNS004
VLV035
ATM014
ATM008
ATM005
ATM013
END074
CNT116
HMF009
PLR004
OST032
OPP001
BLR031
BLD161
EPD088
BLD003
SLD013
BRS005

CTN009
WLF006
CLR013
OVR057
PNC014
CLR015
HYP073
PRS017
VLV031
OVR005
PRT034
ECC007
PPL003
PLM030
CLN010
NSL005
GST042
VLV016
URT013
EPD012
CNT010
URN001
GLL007
LTN004
PRT132
WHM001
DYS211
RNL113
MSC170
FCS002
AGM019
SRC024
MLC004
HLN001
HRM017
SLP004
ANP005
VLV034
EPD003
CTN004
INT082
CMP034
NRL004
ACT064

EXT010
MST002
MLL006
HMZ003
SPC030
MDD015
LRG014
MLT156
CHR619
OVR105
ADN017
SPL012
LGN002
BST002
PRP028
SLP010
CHL084
FML344
GRN061
URF003
CLR131
URC014
MTH076
NRR002
TTZ003
CRT046
IMM199
TRC121
RTN232
DRM050
ADL096
FSH003
PPL004
ACH002
SPP006
BRW005
ATS312
EST006
TRL002
PSD043
CMP066
ENT010
ESP002
PRS062

MCR037
OSS014
CRB191
CRB094
PTC002
SHW006
SLW005
DDN010
GST007
MYP002
SMT002
GRM005
OPT007
THY123
NRD001
LDW001
UNL002
CHR028
GST029
TYR004
HYP034
RMS001
VBR003
XLN110
PPL050
RCR004
NNS045
PLS031
PLM184
ORL019
FRS012
ATS009
MYX005
PLY168
PLY169
PNC085
CLR001
ADN075
GLN002
SMT006
GNG006
ORB012
HRD018
END039

LNG013
NPH002
ENC014
KLB004
FML253
CNN001
ALV005
LNN001
MLD006
MYP106
ICH054
EXT059
INF084
TST021
PRG021
HYP780
ICH069
NNK001
PMP013
PRS025
MYX013
MNR003
WLL039
AKN002
SBP001
CLR019
TRT001
PSD004
VRR003
HTR003
ACT120
DFF020
RSM002
CLF056
RSM003
CNG491
CHR464
PYD004
OBS067
NRM022
EXR007
RRN010
CRC034
SNS011

PRC051
CLB034
BRN139
ALV007
MSM022
CRB194
AMD002
BWN001
MCL062
OVR114
PRP075
HYP555
VSC053
CHR682
CNN010
ATM007
RHM013
SCR035
PRT002
SPR005
NRW001
BRS004
NRV006
HRP001
ATM019
URT031
BSL006
DNT009
MYC021
FLR003
AMY010
OST173
OCY003
LCK001
LYM053
CHR603
THR099
CMP007
NRN005
VSD002
CHY002
PTT048
HPT025
PSR033

FRD001
SLV026
CYC001
FTL004
WLF004
TNS001
GLM008
LNG091
KLT001
BRC011
MLG061
LYM005
CCK001
DPH021
CRN031
HYP144
TTR011
CLP006
NTR055
ANR010
SCN052
IDP067
DSR031
HRD088
ESN022
MRZ001
MDN001
ATM002
ACH004
LPD016
RTC009
CRD147
PDC001
XLN228
LRY002
YWS001
ACT048
MNC004
TRN003
VLV036
CYS015
PLR006
SCL002
GRY001

NSL029
ALT002
HDR001
TXC003
MLT007
CRB001
SZR001
NNT049
FGF014
TCL012
ANT066
PSD088
CNG513
BLR027
ACR116
FCL074
BRT002
SCH079
MCP043
GLC113
HRL003
ALL001
JVN012
VCS001
C1Q001
OCL052
MCL018
DRM056
VRL025
SBJ001
SYR003
NLX003
ATS298
SPL009
RNL025
RTR001
GM2006
HYP078
ATM077
SPC022
INT043
CHR342
CNG515
LNP001

BRN147
MTH086
RRC004
BRS053
CHR465
ADR041
NPH055
PRG019
MSC036
RTN090
TYS001
ALB003
BRD010
LPP002
CNG413
ADP001
IMM165
GGR002
ERL056
DND022
NKC002
THN013
BLN006
BRN031
BLD129
CNT037
MDN002
ANL017
VSC064
FML015
DFF039
BLC004
FLL023
GJB007
PLP007
CNG092
ORL006
TST043
STR046
MYS002
TXC009
GGN002
NNT017
CRB051

PRK046
RRN022
FML156
ENG004
LCH013
PRN061
NTR007
CLR123
NRP065
CLR139
CLR145
WLM014
CRB041
DPY001
GRN063
MYS016
BKS003
YNG002
ESN009
CLR066
CLR117
NRP064
CLR140
CLR141
CLR042
CLR059
CLR125
CLR124
CLR138
CLR054
CLR091
CLR104
CLR126
CLR142
CLR143
CLR144
GRN065
THL003
SNR003
BLC008
DHY002
CLR127
CRD017
CLR053

CLR099
CLR105
CLR090
CLR097
CLR095
CLR102
CLR088
CLR101
CLR107
CLR106
CLR098
CLR094
CLR116
CLR092
HYP603
PRM288
PKL001
CLR134
CLR068
CLR135
CLR136
VNS013
CLR056
SMN001
TCK002
HYP046
PRN015
MSH001
OTS003
DPH004
DPH005
CLR114
ACT202
ALL002
DVR001
UTR006
VRL009
FNG002
SPT003
WST002
ACT018
SRS003
OCL007
SRS002

EPS002
HRP003
CMP033
DPH028
DTH005
HND013
HMT019
MDR004
MND006
CHL098
PDG001
VSC023
PDG002
PNC055
CNG133
CRT034
ADS006
HHV001
MST008
ALN001
TRT019
TCL002
IMM122
HYP802
URT051
PSD063
CLR085
LYM107
CHR320
VND007
TYP054
ATM102
CPL004
CLL002
APP018
BRS001
RPR002
BLD028
PRN022
PSD015
SLF002
NTL004
OPP002
PPL014

OST004
AMP009
TNN002
BNS007
ACT052
SRC006
PST015
ADN013
FML031
CHR177
GLL013
CHR047
CLD007
NSP003
NNS072
SYN106
CRY036
SYS053
CMP004
VNT035
PLN008
PRS122
ONC005
DSC013
MLT118
TRP006
PRC052
TYP050
PTN004
LYM114
SRN001
INF085
ANN002
CNV004
SYN063
ACR088
BCT019
IMM184
NLD009
WLF013
PLM150
ATS307
CNG478
HRP005

STS003
ACQ022
ATR008
FCT008
HRY002
GNG004
DMN001
BNG002
SML009
GNG008
PLV001
JXT003
BTT018
VRT003
FML340
NSL006
BSL013
KSH003
HRD086
MD5001
PRX009
VCN001
RRS010
UNV002
TXC020
ACQ043
LYS029
NWN001
ORF053
PRR019
FLP002
DSR074
NRM021
SNS008
PRP001
SCH072
MCL046
PRP082
ALP041
STR092
ENH001
PST048
FST001
SPN021

CNN002
ACQ009
ATS005
FRS014
MYT021
HYD064
MCC012
SCH076
CRZ001
SMT008
HYP798
INF076
BCL010
PYC001
ECT070
LWC002
PGT008
LPM010
CHR084
NCK004
DRG013
HRT040
GLL029
PRT091
MRG002
HYP018
NSD001
TRC087
GRH001
HBN001
CNT033
CRB006
ECC002
TST007
HYP847
MSC004
SGM002
LCR011
RNL013
BRS056
PLR003
ATM017
SKL003
MLR006

OST007
BLD019
RTR004
SMN005
BGS001
PSR034
TST004
BLD039
ANT005
URT035
FML040
CRM002
RNL016
LTM002
TST010
NNS003
SBC017
GST016
ULC005
CRY010
PRG149
HRP008
MRC001
TRP020
BCK005
CRN175
VSC013
ALS001
MLN066
PRR015
MCP048
MYC079
SYS067
BLP048
HRL004
SSS001
ASP001
ANX007
MLT135
TYL002
SCH080
STF002
DLY008
ATS370

ART103
PLM127
FRN044
MLT095
EPD022
GYR004
EPL113
KSH001
HYP700
PRG106
NRG006
PRV003
MLG007
MCS004
RCT023
ANP006
PLM013
BCT001
CHL035
DRM008
HYP141
MLT031
PRG074
ACT034
ANL004
FTL021
FTL064
CCN001
ANG034
TYP044
MTB016
RDT005
SPS016
VGT002
SLR005
CHR463
VNS012
NNT037
PSD084
PDT041
URC012
NNC012
MLT105
CHR695

PLN026
MYT029
MLN073
CWD006
ACT078
ATY005
BRS049
MLD017
NPH001
LPR023
FBR070
WLK001
MTC146
FNG015
MCP047
GM1004
GM1005
GM1006
TYP037
NRN008
CLR079
MGB001
ADV001
MSC047
CHN055
ALZ031
PLY167
ACR091
PRG092
MYC055
NRP066
GLY004
CRT086
CMM003
ANT011
CRB025
INF146
SDR009
IMM187
PRS013
SKN018
BLD052
NRL001
PHL003

MCP004
LNG111
DFF007
KLL001
PRG010
ATS491
CYS042
AQG001
CNG024
CHR055
CTS012
BRT037
HRP038
SML040
FRS004
RRH015
SRT004
ATM088
URC013
CBP002
FBR085
CRB087
SBC025
PNL012
MLG163
HMP029
AXN001
FCT001
INF118
SVR055
CRD007
BNB001
THR011
OVR041
VGN016
PHR004
END036
CNT109
HYP507
ATM009
ATM015
ATM105
INT084
ATM018

PRM023
LYM023
BRS046
CLL012
SNS026
PRT119
CRD086
MLR023
PPL048
CRY030
BRS002
TYP028
SLV025
RNL115
RTN150
THN009
ANK017
DFN308
SPN030
PTC001
TYP036
ATM097
MNK003
ATM092
IMM105
ACH043
GMM001
XLN230
MTC059
CRZ002
BLP051
DFN182
FLL041
MCS006
RNL122
PRM025
CMM006
ANT003
ANR001
BLD040
GST048
KNB006
PLM149
ACH041

CRN239
CTR122
HYP059
IMM149
CRD184
THR117
CNR003
IMM095
IMM074
RTN160
BRC081
HYP210
HYP741
END081
HYP744
CRN278
JCK001
HYP042
NLP001
OST044
OST131
PSD014
TYP056
RHB008
ZYG003
DRM041
OCL039
RTN130
PRS035
PRM026
ERL002
RSP005
BNR001
BND014
BND004
SPN250
ACH011
CRD217
LGM010
ISC005
BNS002
CFC003
AZT006
PLL016

CHR679
1CH001
ATM098
CHR565
CHR569
TMT006
FRT005
BTL002
PRG147
BLL002
CRN034
VTM036
OVR121
CNG357
EPD017
EPD014
SPN031
MNS001
CNN004
PRN002
CHR009
PLS010
CTN010
NSL022
VLV021
ANS012
MCN003
PPL007
OVR010
SWT008
HPT020
CNT017
WLF003
RCT008
MLG067
MLG076
OVR060
OCL009
46X057
FLL029
VGN018
HYP028
END035
ECC008

INT379
INF162
JJN007
MCN021
MLR007
MXD010
BNM001
BRS057
BLD043
URT024
EPT010
ECC003
LNG017
LRG017
SBN003
LNG023
THY026
CLR014
EPT009
EXT023
DRM005
VLV009
SCH001
ADS003
OVR054
CMP001
PDT001
ADL001
LTR007
CHR082
CRM013
ONC001
UTR042
VGN015
MCR014
LNG019
URN005
SQM004
MDS011
MLG048
FML039
RDL003
BLD025
AMR001

BNG001
PRP021
JJN003
SBG006
ATN003
CTN002
GLM001
ECC005
APC004
PRS033
VGN012
EXT035
KDN016
BRS031
INF036
ADM001
BRT016
JJN002
ORB002
ORB011
INV003
OVR011
MCN004
RTN015
ESP012
BLD032
NPP008
INT380
SPS002
BNG021
PRL002
KDN025
GLL051
EXT020
CCL002
ATR007
MBT001
PLR002
OST014
TRC018
RTR005
CPL005
URN009
INT049

ECC001
URT003
BRT060
RNL017
CRV037
ANL006
LTR004
APC005
BRT015
CRV014
BRS025
CMB002
PLM004
UTR029
SQM005
SBR003
MLG036
INT041
BRS013
URT028
PNC002
PRT021
PRM225
NPH008
VGN010
ANL009
THY018
ATY004
UTR037
CLC009
TYP042
LKC003
IND017
ADR012
LCH004
MTR008
THY003
VLV030
CNT031
MXL005
KRK001
FBR013
SRC009
BRS030

CND008
NND001
ODN005
INF025
PRM126
ANR006
PLG004
RDL002
ANG025
PGM030
PDT035
SBC039
CLR112
JVN038
SLT009
SPT024
CHL109
MTP005
PHH001
WST010
TBR012
EXT064
DFN344
ECT102
EHL079
FML346
MSS001
SHR109
PLY162
ATH001
TMR016
PLY161
CLC046
CNG193
SZR026
RBN014
JBR020
ORT011
ARM010
CGN007
SLT014
LKD017
ATX024
PLM121

HYP535
PRM056
PRG080
PRT035
PRR004
MNC019
CTN011
FNC005
INT074
URC002
49X006
SDD004
ATX010
HRD084
ZLL002
ESN004
NGH001
CLF004
CHR579
BSL038
MLB001
EHL052
HMP032
LKN026
THR110
AMY113
TKN001
SLR001
INT319
CRN234
HRM021
NRP051
ENC039
RSP008
GLY043
RDD003
HML042
SYN088
GST111
STR007
CHR096
MCC004
ATM082
CTN005

AST002
ACC005
ATM061
PNM022
MTR086
NDL021
XNT009
INF175
MDR006
FRT004
OCC011
MLG157
PLM028
RST002
NRT011
ACT098
RBS002
SPR006
ANS003
CRN022
CHR059
GRN020
ANG037
IDP047
SCN082
FCL041
IRN004
PTT009
PRN029
FCH001
BLN002
MTH007
NDL013
KRT063
TRN053
MYL020
TLG001
BWN006
PYT001
ISL119
FTD001
ATM064
CCT003
PLX004

RKT003
LKP001
SKL032
TTR001
GRN010
OLG002
PYS001
VRR004
SYN031
BCL011
CYS002
GRN011
BRX001
DDN009
PTT004
NCR009
GRN004
MYG005
ACT028
CSL001
SPR019
RTC003
DMP001
PYL006
FBR032
FML355
IGG015
FCS014
TRC010
PHL001
RCK002
SRC011
DYS011
RFR013
CRN233
OPH001
TQP001
SPP004
NCR002
ENT003
SKN023
BRN055
RRS004
HYP810

MLG141
MSC162
SLT007
SPN029
MLT075
RNL012
DBT018
IMM206
FXD003
CNG048
FBR028
END020
CLB010
RTN209
HMP004
ATS013
HMN002
RPT005
SPN011
PRP013
CYS014
ATM076
EXF003
CNC014
MTR004
PLY170
MYP138
LRY029
SPN392
AMY087
INF088
BRD003
CTS001
GST030
MNC001
SMT001
ADN009
CLL014
CNG112
EST005
MYX011
CHR692
DNT046
BLM002

PRG020
EST001
HYD038
PLY131
HMF008
ARC025
HMN036
PLM026
GNG025
BYS001
PTN001
CHN010
LKC002
RFT001
CPL006
PRT014
LBL001
PLY021
PLL002
HYP706
PCM002
RNL078
RSP018
CHR280
SYS066
PGM010
LYM150
DVL012
SPR086
PLR025
AMY003
SCK002
ABS022
HYP121
IDP023
EXP002
HRD039
PLY187
PRV006
FBR084
INF078
ALG028
NDL001
CHN002

HRN001
NNL001
EMB005
RHS001
GST020
PTL001
INT009
OLC001
ADN014
DNS007
MLN046
SCL025
WST009
CLL026
CGH002
OVR077
OST006
SPS226
CRB175
HND015
GLC096
CRT089
PRM032
OCL002
BRN133
CYS013
DRM014
FRB001
PMP004
LYM012
MNK002
HMN004
HRY003
LCN001
PRS011
HYP021
BBN001
KKC001
PRN032
SLN006
BNN005
TRR003
HPT070
ESN012

FML091
MYC088
VRL017
DFF010
LRY022
TRD006
PPL054
LPD026
NRF023
ANS021
LBR036
APP003
CRB027
ANT019
FLL031
ISL001
IND004
LYM008
LCR001
GLS001
PPL018
PSD003
CLB002
FNC004
FNC002
ANC001
BNG018
TBR009
CRC001
PSD016
CLS032
ALV003
HMR014
JVN042
RMT001
PTY004
LYM095
XLN247
FML292
ACT181
PLM186
ATM111
OMP004
SPL004

KRT010
BNG091
VSC044
DRR014
DNS009
PTZ001
BSL037
HYD046
ABN011
SCT005
PRM001
CHL140
PRM093
ANM035
CTY001
TBL029
YLL001
HYP801
PRL013
IDP043
DYS007
CNJ017
BRD011
HMN001
SBM002
PLS025
DNT001
ANG016
PLC001
NCR003
DYS009
HYP009
MLL002
MLG142
BLD049
DNT007
RTT001
INT062
CNG486
BNT001
ULC007
UNC014
HYP048
PRM163

CMB089
SPT007
EXR008
PRM340
MCL038
VNH007
AFB002
PYR039
ALK019
BSL036
PLY006
IMM221
MJR021
FDB001
HYP794
RNL100
THR032
MTC003
CRL006
ACR062
CLR077
JVN059
ALP087
INT047
ADH006
LST003
MCL001
CRL004
TRN004
EXC003
VLV032
FCL001
CPL001
HMN009
ADL052
PLX002
DFF019
FLM001
ESS001
GST010
PLM011
DSM004
CHR054
XLN245

DTR001
BRC002
GLL017
OVR012
ATS382
PRX001
HSH004
LM2002
ACT231
HMN016
ODN006
TMP006
THN005
MTG001
PRQ001
PRF003
CRT033
EGL001
EMB007
GRN032
JVN060
NKD001
HRD224
RVR002
TRC096
STP007
INF184
LPS018
PRN071
HYP855
ENT007
JCK003
NNT018
THR035
EST002
APR001
ALZ060
AMY088
AGR018
RNL065
FCL049
BLD145
CMP028
EPD083

NVC001
DBT091
FBR052
MCP049
GLY008
SNL009
PLY183
CNG006
BRC006
ADR016
FSR001
TNP004
ABT001
URT020
CRD065
INS023
ENC038
AVS006
BLD045
NVS017
ORB019
CNG010
INF151
CNT026
CHR418
CHR020
SPH010
OVR048
INT025
CTN033
DYS003
HST004
INT003
BLD047
HMG003
NDL009
TNF001
MXD026
RNL097
ACT053
SNL004
ACN003
AML001
MDD003

HYD001
ACT046
MNN007
TRS001
DNT010
LCT003
CHR095
CCT001
ORB007
EPT012
OLF005
CNT025
SBS004
PLY024
VTR004
SPC005
CRV025
PTY001
OPS006
HYP055
TTR005
CHR176
DST107
ERL029
IDP033
IRD003
MCR039
PTT041
ENT016
BRH001
WLD005
HND011
EPD090
TCH005
ADL080
ATY022
CMP102
MLT116
CNG124
LGH004
MNG006
ADG002
CML001
IGG010

CRY031
PRS120
NRN047
SCL047
INF087
FRN051
SYS038
FML023
BLC015
BSL009
HYP003
PYL018
IMM166
BLR028
VSC063
OSS012
THR067
BTT016
LGH007
CRC014
OTP006
ALP039
ANK020
MCH002
WRN002
OPT051
DST033
GRV009
DNN001
DRV001
CHR515
MCR312
CRP030
ANT009
CRT069
LSS002
ALC001
TYR013
PRK022
BRT005
ADR042
SCH038
TRG006
PLM167

STF001
EPD052
EST007
CHL116
EPC005
NPH049
CTR121
CHR593
LSN002
ATS383
HRD144
MLT093
VRT001
BLD041
PRS144
SCL014
LNG116
FCL022
BLP004
THY006
RCH001
HMP003
PYL001
ACQ010
KDN001
SBC009
ASC004
CNJ018
PNC012
GRN055
BNS004
ACD008
END007
BSS001
HYD003
ENC003
NML001
PRV005
ANT001
VRL006
ACR014
TST018
FNC007
SLC004

CRN009
MLT016
ISC001
IMM002
MGL001
HYP050
ULN001
CCM001
JVN008
SNL001
MCC003
END028
SCL008
THY009
NNN005
ANG017
PMP010
HYP057
URT016
OPH002
ESP001
KDN015
CRH002
IMM044
NWD001
ATP014
EPS038
IRV001
PST041
UTR043
ATR081
PLY065
PNC049
DXT002
IDP090
RRG043
NNF007
VNT036
EXT062
LRG008
PNH004
ZNC005
IDP079
ADN072

MYT011
MLT084
FML307
EPT020
LNR009
GLY081
ATM063
INF124
MTH047
SYS052
CTR077
ATR001
CHN065
LPD021
CRV043
CGN001
DWL001
WLL001
ALX003
ANS023
ATR089
AST054
CRB011
CLB022
NNN008
PRX015
STR084
MCP044
GLY016
FCL009
TYP049
PTR032
NPH013
PSD107
HPT085
THR125
KRT073
INH001
BLV001
ADR054
BTT001
VRG001
CRB176
SPC026

PPL058
GLN011
CYC008
FNC046
INF092
LSS005
SCH074
LFR007
CTN028
BNB002
TMR010
ADN024
OLM003
HMP023
SPN294
SPN209
LFR001
MTN003
PCT003
LKD025
HMP013
DMN005
NRD008
CLC045
FTL002
FRY006
INF083
CNR001
RSS001
ANT088
DXT004
SML016
ANK021
BRS050
PLT006
MTL007
NRG001
MYR001
FCL007
PRT012
NRX001
GNG005
BLD053
STR021

PNM003
CHR546
GNG002
GRS001
LTR002
SGT001
OCC001
LNG030
DNT003
DFF002
MLG074
PST014
SPS004
HYP007
SPS005
ACH003
CRV040
AGN002
FMR003
EXT032
ADR004
HLP001
HMT001
EMR001
DGN002
MLD002
TTH004
PTL003
BRD004
LNS001
TBR003
CHR017
KRT079
DDN003
PRT015
BLT003
CND002
NNS006
SPR017
CYC002
ADT002
AFF001
RHM008
PLM041

SPT002
ADR008
CHL033
RTN011
AMY002
ISL099
NSL028
DXT001
PRL018
CCC003
ICH018
NTR011
ELN001
MXD014
CNG216
DFF021
GLS013
LNT002
RTB001
XNT007
FRS019
PRN068
UNC016
CVR008
CNT101
PST037
PTY006
PLY039
DMD001
ESN011
INF057
MYC018
EPS023
SNK001
FML337
PRM248
LMB076
CRN309
MLG164
SPN340
AMB007
CRT088
MCR103
PRS110

ACQ027
SYN116
PLY138
ACT235
CNG506
PMP008
RRH030
ACT236
EXT039
FNT004
IDP038
PRM304
INH023
PRL048
RHM032
PCS003
UTR054
PRM301
PLM189
RCK009
INF089
CND005
HMC035
HYP540
MGR025
CRB193
PRS108
SNT005
PLM007
ACT149
MCL075
NRP054
AMY086
PCH015
ANT081
PRT008
EMR018
SPH013
KR002
EMP011
MCR247
NRB010
STR089
THY102

BRN019
BLP046
CRD187
ACR012
PSR024
DFN136
MNT311
AML066
TRC110
TRM004
HNT010
CTR177
TNM001
XRD030
URG005
NTR036
HYP609
NTR013
EXN001
NVS015
LNG105
MYS019
HRM008
GLY015
INF091
MYP035
SPH014
FTL071
IMM239
SYD002
TBR002
LMB002
PNC005
TRC006
TTH031
LYS003
EBS001
TRC092
NPH019
LYM139
INF159
HTR021
SPH015
TRC038

MST021
EPL200
BLD155
HRM006
PRG038
IMM038
JVN004
BRN062
TKL001
PRG040
PLM156
HYP818
CMP063
LKM056
ANL019
BRN129
BRS109
EPD002
MRL006
FCS011
HYP737
VBR001
ERL050
GST103
PRG130
TBL031
PNC118
SPL067
SPN405
THR071
OST122
DFN159
CRN277
INF090
CMP042
CTR181
MSC181
CLR075
SYS055
CMP089
AMN014
MTR020
MTC114
RTN046

CND001
EXD001
TTH002
PRN020
CTC001
SPT022
ATM103
CWM001
TBS001
LMN013
PRS064
MLT094
CHR563
TRC034
ANK022
BNG013
OPT001
INT012
HPT081
CNV002
SKN013
PRG001
MYP001
DYS006
HRD010
PLM009
VSC006
PLN001
TNS013
HRD026
DST103
ISL078
OLG022
MXL008
NRS001
ADJ001
AXL002
BLT001
PLC011
SCL017
ESP011
DYS005
FCL010
CLR012

PLM019
MCN011
INT042
KRT003
IRS003
BLD009
ALC011
CRT012
TNM002
HMG001
SRC001
DFF016
LCR006
BRN035
CLR017
ERL001
VST004
NRL002
GLY031
SPR007
GRN001
DFF015
DYS016
TRN007
GST013
OBS003
RCK003
BRN145
THL010
SPL005
SRS004
VCT001
THR007
AQS001
PNT003
LPN001
BRW004
CRN016
CRB036
MDS006
MDS019
PRN008
BLT006
CHS005

GNT005
SBV001
WTR001
BRR012
ABD007
AMN006
PLS016
SDR003
ZLL001
SBC005
INF018
IMP001
ACT056
GBL002
HYD015
NDD001
ULC003
LCH003
BRN053
HRT017
LBR003
LRY028
MGC006
MDL009
NKJ001
PLM052
NRN016
PDN001
RNG019
THY042
URC003
CNT075
GRN005
CRD006
RRP001
ATS411
ERY002
CNT068
ORB016
HYP047
PCH002
CTN016
CTN027
HPT028

MNN022
HRN003
ARW003
ASP009
ATY012
ATM074
ISL121
INV015
AML065
PRM155
PHC019
PRR018
PSD047
SND005
WBR001
PRM314
THY089
ELS004
ACY011
CHN053
IDP093
BLT023
CYT017
CLM004
ESS005
LMN011
JVN030
LVT001
MLL004
MRK002
NNS011
ONC003
WND005
CNG609
MCL047
TRP014
SPN050
FLL042
PRX097
NRN022
HRD112
BCT018
JVN033
HPT066

RRV009
PLS037
RCN002
HVY001
STT041
CRV068
CLC037
NTR049
HMM004
SZR029
KFR001
GLC084
ALZ056
HYP139
BRN034
ATX023
MTC027
CTR157
FNC029
ANS018
OPP004
TMT002
DRM058
MNC002
FRG008
PSD008
PRP069
VLC001
ACH015
ACT238
DST008
WLF002
NTR047
CRD186
ART001
CHN044
CTR172
CRG003
CRD221
MRT001
BTH005
NSP009
NVS018
OPT070

OST135
SZR022
CYS041
DYS096
ECT100
EPD029
SND002
RNL024
FCT004
HMC040
MCL016
CMD004
CXV002
DYN002
ART028
HNZ004
HTR005
SPR004
SRF011
TRS030
TTH030
TLN014
GLY003
MCP050
PRN038
PYL012
PYR041
SVR066
ATS366
FBR099
KRT038
ALZ050
MYT020
PPL035
NXS001
IRN008
CTR176
HMP014
SCC001
TRD003
IMM099
CNG184
ATN023
GLN001

TYP004
HRD007
CNZ001
MTG002
OTS008
CTR144
CTR145
RTN186
PRC054
ACT240
PRT136
NRD106
LKC005
SPR088
LPD035
FTL075
NPH032
ANR046
ERY062
CRP037
IMM237
ART035
PLM170
PLM070
RTN072
RHB023
PLN017
FRG010
CHR081
ALB021
CRN106
CNT097
KBK002
CRD093
CTR125
CRB174
TRC077
PGM007
CRR002
KLP010
KYR001
HMC041
DBN001
PSD106

NMN013
NRD017
HLT002
CHL134
JJN004
LTT002
MSC037
SJG002
PND002
BRJ001
BLC001
STR081
ATM053
SCH045
SPN296
PSR029
CRD166
STL007
LPD019
IMM055
INC015
ACR084
NPH076
TST025
CLC047
CNG411
HST022
OTS012
NPH035
HJD001
HYP679
ANG001
ANT041
OST080
HPR005
HYP748
ELS002
CYL004
ERY053
GST095
HYP732
HYP864
HYP293
LPD015

TRC072
MLT159
SPN226
NPH031
BRL011
SKN065
GLY005
ICH020
PRS134
TLM001
OGC001
SYS047
ACR090
PSR030
XRD032
AST057
ISB001
CVT001
WHT020
FCT002
PRK005
TRP008
HYP097
INF049
ASP036
ADS001
LFT003
GRS003
ISL003
DSC001
PRT030
PLT001
PRX005
BRN143
IDP006
SCN005
CLC011
SHR030
FRN012
DNT002
SPN048
ENM002
INP001
HRD001

CGN005
DYS018
ATS331
ATS217
BRD014
BRD016
BRD021
FML270
BSL004
EXD002
ACT016
MDD009
PST020
MYB001
PLM025
CLN009
MDD008
LPD007
INT020
PHT003
LMY003
SPT001
OVR058
PRC010
ECT003
RTR007
BRT012
CHR076
MYL002
DPR002
URT030
PRS002
PST109
MYX007
ECH002
MSC072
LFF001
CHR043
TTH007
PHT004
ANG013
FLL004
LTR003
SRC002

SKN001
URM001
SPN036
DNT011
RDL004
PTS001
INF022
FRT001
THY001
FNG003
DPH006
TXC004
CNT018
SBC014
NDL006
DDF001
ANS004
ATR073
URT014
CCM004
RTN002
PRL006
TRN012
PLP002
GNT004
INC033
ATS483
SVR107
SPN006
ATR009
CHR018
CMP006
LBM003
ESP017
VNC001
GST028
LPD003
CMD001
EMB006
CNG027
EMP001
THL004
SCR016
AMP007

MLG070
ANT013
MCN005
ALC012
INF005
MNN004
CHL040
SKL005
BSL008
PRM014
RCT005
ADL066
ATH004
ATT003
BRR004
TYL001
MTC004
LVR004
PRL005
PRT009
MLG056
HRD016
CLS034
GNT023
ICH014
MTC023
NNN007
SHH001
CHR067
GRN059
HRD104
STT071
BLR012
BZR002
BNM005
SML041
CHR287
EPL230
ERY018
FTL012
HMC012
IDP022
LRY027
ERL012

MCL022
PRM243
SNC001
TTR012
URT040
CYT019
GRN060
RRM006
TRN046
PRM133
HMP028
PCR003
OVR073
PPL026
PPL027
PLL009
LNG015
CNG129
DND018
DSS012
GST055
GRV012
IND005
LTT006
LPS019
MSN011
MYH016
GNT044
NTH004
ZLL011
BNG042
BWN003
PRM299
RRH004
RRR002
FCL090
LWG005
GNT037
CRB132
EPS047
FLL045
DFF029
IDP078
NDL020

MXD051
CRN311
RRP028
PRG033
RCT024
RTN235
RTR012
SNG014
ZP7001
NRN023
ALK017
EPS044
PRD026
MYS028
ETH013
VGN031
ADS015
MNS012
LYS030
INC036
BNG081
ACT161
IDP073
PRM150
RDT017
SCN066
HYP854
IMM236
CNG101
CNG418
CNG439
CRY006
FML364
LMB074
LPD038
MNR004
MSC026
NNH002
OGL001
OGC005
INT110
NNH006
NSL020
FRS011

PRM158
PSD079
RHZ007
VRS001
DMN012
BCR002
INT176
RNL123
PRD021
QLT011
CHR688
HGH021
ALK016
NNT042
SCN051
MLT131
SRN003
INT189
BCK018
RCM001
PRG003
ORG002
MTR024
LPR016
ALN005
GMM012
INF081
MYP099
IMM151
CMB082
LKD026
AMY111
ELL006
PLY181
LYM151
MRR015
MCC013
HYP807
LTH002
BRT055
SPN251
ATY003
CRB028
ALP005

FNG001
3MC003
MYC083
MLT072
BRK001
NRP063
PSR027
CRN174
CPL014
SYS051
SCK017
MND002
PRS135
LNG073
PST036
MGL009
GRW026
MSC176
OPT048
OCL066
SYS046
ALZ015
GLY010
NPH078
PHC018
ATS406
SCN049
PLM196
PNT019
ATS204
HRD021
RTN032
RNG030
ARR045
ART143
VTR016
ACY010
CRB160
CRP032
DRM043
TRC086
LCT013
DYS045
UTR057

WRD032
CHR105
MTC056
LBR004
CRD183
OTS013
CHR637
CYS044
LYM145
MNL001
MYP125
MYP087
HYP370
MLT160
MCR364
MRR011
DFN341
FLR007
MTR019
ERM002
FBR088
SCP012
SPT006
SNG011
PRL045
PLM059
CMB022
JHN001
MND003
MTC026
ULL002
CRN294
MYP091
PLT031
PRP091
PST040
CLB019
LYN004
CRN238
CRD002
DNR003
DYS053
DYS191
ELL005

BCK002
CMR001
EPP017
SRS007
GLL036
GLM012
HMN035
FTL033
VNB005
LRN001
MCR237
MNN019
MGC002
MCP052
SPN394
MSC169
GLC012
NRM019
STR039
SYN084
TLN006
TLC001
FCT003
GCH016
GCH017
HMC038
GLC097
MNS015
PRM196
SPS133
PSD108
ALP096
ANR033
IMR002
GLY033
PLY158
GTL001
ERY048
GLT007
ATX049
CLB017
HYP870
CLV004
CRV071

CRD097
CNR007
ACR041
CHL136
LSH003
PRT125
CHR516
TRC095
CNG189
HYP332
PGT009
CRN247
ACR011
MGL030
BSM002
CHY005
SCH018
ADR023
ENC054
RDC016
AML061
ISL122
DBT090
CRN221
PRK025
PNC094
NRD069
HYP859
SPN228
DFN382
GLC114
SPC010
ORF002
LPM007
AST035
MCL030
SPS153
FBR068
BSL028
PRN001
ACN016
RTN159
MNT263
PRM252

CNG504
CHR542
NSP015
FRN048
VRV001
MTC212
BCH004
KNS007
PLY166
ENC010
FCL028
BLC012
MYS014
PLS029
MNN028
ECT045
HMG032
GLC022
HYP110
CTR160
DVL067
IMM201
RTN233
SPR020
ARG001
INF006
ERL057
PLM151
PLM182
CNG208
RNL028
46X079
VSC014
HYP559
DSN001
XRD022
DNT020
CRD031
IMM026
NPH108
VNS015
OCC006
SPR119
ASP002

ATR002
FZL002
CHL132
CMP072
DST005
DBW001
CRB185
BLD169
BLM001
BRN076
SYN086
PBL005
PLN006
PLY137
FML345
JVN014
PRK080
KRT029
LRY046
MGL003
MCR334
HRT031
GST093
LMB010
EPL118
MTP003
MTH071
OST164
DRR017
SPR089
SPN401
HYP795
ALP099
KRT057
TTH032
DYS064
SCR020
CRB188
EPP012
ABC001
THR037
PLY052
CRB150
BRN077

PNC111
MTR044
ART105
IMM178
MGL012
PRT130
FCL043
GLT039
IMM078
CDL005
CRN111
MLN043
GLC105
BSL034
CHL142
DVL118
IMM071
MCL078
RTC008
IMM075
VLT001
ATR026
NRP048
MYP044
CTR159
OST168
INC031
CLD014
MYP071
CHL114
RSS025
PSD093
HMR039
HYP547
HYP523
IMM096
IMM182
ART107
HYP578
CTR182
IRS011
ANG060
AST032
ALK020

SHR068
CMP095
SMT018
IMM064
SPS103
LKN008
ACR092
PTT045
DRM060
DRM061
MYP022
IMM153
CND037
ANH003
AMY082
AMY060
ANT078
APL023
BLD146
MTP025
HMF006
GNT026
HYP736
HYP364
ICH073
HYP186
HYP160
EHL034
MLT152
CCK007
HYP652
CHR211
MRS002
MST022
NRC009
PRG018
PTT016
PLG001
SPN301
PRP038
THR017
BRG005
TMT001
PSR026

ECT007
FCT006
ACR122
MSC179
OPT025
HYP793
BRD018
CTR110
DRM055
PHL006
LNG062
PLY068
HYP841
CLC048
SYS069
MLT060
SYS048
HTS002
NRD009
DFN178
OPT024
MYC054
MSC103
CLR080
SYS040
INF079
OST136
THR068
RTN043
TBL022
TLN011
PLY171
EPL140
MSC177
SHR097
SVR096
ANS002
ORP002
OCL001
VTL001
SPN423
STR086
EPT025
PRM030

RTS001
ATS384
HRD201
MLN079
SPN221
MYP108
UTR056
NTM002
ECT093
LTH003
INC035
GLP001
MLT177
BRW002
ACQ057
SCN048
PRD017
BNS001
PDT026
PLZ002
ATS385
MTC010
SLT016
SML005
APP002
BRN144
INF194
ACC002
DGN006
ANK023
NNP021
SPN444
ADL100
SPR165
PNM004
SCN003
FBR016
PRS049
FML286
FML361
HYP062
CLN011
MLT010
DSS010

IMP006
PNB001
PLC006
ALX001
NRS002
NTM001
PTH001
ACT001
WST003
HRD012
VLV018
MLG005
CLL001
MXD027
RTN006
LVR002
RCT009
HYP070
PHS001
MTR007
LNG037
GTR001
HYP029
OPT008
SBC002
CYS011
BRN021
ALP012
PNC103
DSR090
HYP085
PST007
PHR002
ULN002
RDL001
BRN120
TXC008
KLP003
HYP026
ATS277
PHR008
LPD036
BRD017
BRD020

BRD013
TYP058
CRD178
MCL057
ALP072
ATS015
ATS018
CTR185
NRD007
ACQ017
CHR286
TWN001
FLN001
JPN001
ORP001
FLL006
SCT001
MSN003
NMN001
VSC004
EMB002
MYL075
SBG002
PLM020
CLR007
PTT008
OVR047
LRY011
HRM002
VLK001
CLL004
RTN013
HMG004
BLR005
SCL004
DFF004
GTP001
PGM002
INF027
BZR001
CLL005
HPT006
SLC002
JVN002

GST041
BNG019
CLV002
OVR031
DBL002
PST022
CNT019
PST025
ANR005
KHL001
PRM293
LYM157
RSP023
LNG115
PNC007
CHL002
ACT031
SQM003
ADH001
CNG003
LCR012
BRS061
HMN003
OSS002
LYD001
ART003
LNG035
VNS002
INT013
FRM004
PRS019
RNL004
URT032
CRB030
END031
MRB001
CMM007
CRB024
APC003
PRS044
ADR005
BLD038
NDL004
CHN003

LND001
EXT009
KLV001
BRN005
EXT012
PPL009
PNC039
OSP001
VSC001
VRL001
BRN036
MST001
ORF001
MXL017
EXT029
BNG004
IND002
MDD001
STR001
NNP002
NTR002
PSD005
VRL004
END034
PLS001
RGL001
VTR002
PNS007
OCL011
HYP039
FDL001
PRS014
SBL003
SCH011
LPM002
PRT024
QNS001
STR012
ACT039
PRL014
PNL013
ACT023
VST003
PTL002

CND003
NCL001
MRG001
VLV015
MXD019
OLV001
CCM002
CLN005
PSD001
TCK003
CCT004
CRP004
INT053
NNT010
AFR001
HYM001
DPH003
DLF001
BLL005
ECH001
DYS004
PTT002
SBM003
PST035
ART015
ACT065
DYS017
GST008
SPR018
SBN001
CLR005
ALK003
UTR024
CRN021
CRB022
MLG062
INF011
CHR573
SRT002
RHB001
DST001
THL002
GLM004
BLD030

MXL004
OLF002
SYN004
PRN018
GMS001
VRL002
WRD001
ANP009
CNG004
TBR007
CVR001
BRT004
TYM002
OMS001
MSC012
APC006
CL4001
CSF005
CLD004
NNS043
ACQ041
ACQ012
ACT193
ADN026
ANT089
BDR001
VSC009
OST008
URT011
HYD004
VTR005
PRD002
STP002
BLD008
LPC002
ESP010
INT038
INT050
CYC005
BRT014
RTC005
CGT001
GLC036
HYP705

HYP707
HYP213
HYP137
LFT005
MYL074
NRL008
OLG006
ISC003
SBS005
ACT250
CNT001
HMM001
TRT002
ACT215
ANK019
AN5002
AP4001
ATS309
FML324
GLC024
HRT036
LBR033
RP6003
SLC035
TFR001
BLS007
CLC010
THY008
LNG004
ECC004
SRC004
CLN023
CMP035
CRN305
CRM010
DBL004
FBR022
FBR024
GRH005
HYP194
INF044
KLL002
KPS002
LCT018

MCL079
MYT019
MTT004
MND005
MRG007
PLM062
PLM068
PLM069
PNC060
RCH009
RHH001
STC016
IDP085
DNT004
RDC010
RFS002
RNL034
SCK034
SPN092
STR029
SPR032
THY054
TRC064
WND002
ZSK001
INH007
RRV002
CPP003
RCT033
MXD032
ACQ053
PRM127
PRM129
MYP060
CLC064
LWF001
ACQ056
PTC005
NRN046
CNT115
SRP007
OVR125
INF120
NVS007

CTM001
PCT001
ORB014
PTY005
PLM049
PNT023
ORN004
PNS014
PLT008
PLY036
PLY040
CHR227
DHY008
DBL008
END051
EXR004
FML168
FST010
GST107
GRN022
HYP835
INF134
INT104
LTH004
LCL011
CLS054
DYT002
G6P001
MPV002
MDM001
PX6002
SH3001
WSR001
ZP7003
ACT114
ADL068
ANG033
ANN014
ATY016
ADT009
BNG041
BRT047
LNG081
MNS008

GNT082
INH022
ACQ054
INF115
CLF049
CMM016
ISL135
PRD043
BNG072
THY085
UNC021
KRT077
DFF038
CMP062
ANT063
CNG578
HRD173
MCN018
DPD001
LVR032
ACT241
HYP641
INH031
THY108
STR111
PRG036
RDT004
RCR003
SWY003
SYM005
YSH001
CLR033
FML348
RRV005
PDT048
RRV006
PLM108
FML279
QLT041
LMN005
VSP001
CNG284
GRW021
DSR057

AHM002
46X071
HRD215
SYN122
ISL112
ETH012
ORL022
IMM212
ANM077
CCN009
PRP104
MTH049
HRD216
RHM037
INF185
PRT256
INT381
ITR003
SPR154
FML205
ELS005
HMG026
LYM117
PRM151
DSR026
WLL022
ONY005
CMB069
ACT248
TRG022
RDT014
NNS044
PMP009
CLS049
RHM033
JVN041
RGH006
ATR055
EPB002
CHL076
CLB009
CNG108
CNG441
CNG121

CRN051
DPL007
ESN021
FCL042
FML327
GRD004
HPS001
INH011
ITC001
KNB001
LNR010
LNR004
LPB001
MGL013
SHK001
SYR007
PRK047
RRC003
SPR064
ANG062
RRM003
EXT063
PRG076
PRM227
PRL042
PRP033
PSD026
SCR024
SCN061
SMT007
TRP010
TRC052
TRT022
SCH017
ATM078
CNT067
CRB089
HYD034
UND001
INT276
SPR145
HRD171
ISL036
HML054

ISL040
PRM142
PRS140
PRN069
HRP026
CMP098
MTC196
PSD105
WHT012
APM001
UNC023
GNT148
CLR137
CNG530
FML223
IDP087
WRT005
CLS052
CNG613
IDP095
BLL015
BLL012
BLL018
PST107
ANM075
ESP040
SLT015
