# Supplementary material for: Bioactive Compounds and Signaling Pathways of Wolfiporia extensa in Suppressing Inflammatory Response by Network Pharmacology
Source: Life (Basel). 2023 Mar 27;13(4):893. doi: 10.3390/life13040893 (PMC10142087; doi:10.3390/life13040893)
Supplement: Supplementary file 1 [file life-13-00893-s001.zip › Supplementary file 5.pdf]

**<u>Common genes between inflammation related targets and compound related overlapping genes:</u>**

42 common elements in "DRG" and "CROG":

PLA2G2A
CNR2
LCK
PTGER3
PTGFR
GBA
PLA2G4A
PTPRC
ADORA1
PARP1
FABP1
PTGDR2
LTB4R
CNR1
MAPK14
CYP2C19
EGFR
F2
F2R
FGF2
FLT1
SIRT1
ALOX5
HRH1
JAK2
KCNK3
NOS2
NOS3
NPY5R
KCNK9
PDGFRB
PLAT
PLAU
PPARA
PPARG
PTGER4
TLR4
TRPV1
TRPA1
CD38

PTGES
NR1H4
